# Hospitalized Premature Infants Are Colonized by Related Bacterial Strains with Distinct Proteomic Profiles

**DOI:** 10.1128/mBio.00441-18

**Published:** 2018-04-10

**Authors:** Christopher T. Brown, Weili Xiong, Matthew R. Olm, Brian C. Thomas, Robyn Baker, Brian Firek, Michael J. Morowitz, Robert L. Hettich, Jillian F. Banfield

**Affiliations:** a Department of Plant and Microbial Biology, University of California, Berkeley, Berkeley, California, USA; b Biosciences Division, Oak Ridge National Laboratory, Oak Ridge, Tennessee, USA; c Department of Earth and Planetary Science, University of California, Berkeley, Berkeley, California, USA; d Magee-Womens Hospital of UPMC, Pittsburgh, Pennsylvania, USA; e Department of Surgery, University of Pittsburgh School of Medicine, Pittsburgh, Pennsylvania, USA; f Division of Pediatric General and Thoracic Surgery, Children’s Hospital of Pittsburgh of UPMC, Pittsburgh, Pennsylvania, USA; g Chemical Sciences Division, Oak Ridge National Laboratory, Oak Ridge, Tennessee, USA; h Department of Environmental Science, Policy, and Management, University of California, Berkeley, Berkeley, California, USA; i Earth Sciences Division, Lawrence Berkeley National Laboratory, Berkeley, Berkeley, California, USA; Harvard Medical School

**Keywords:** human microbiome, metaproteomics, microbial colonization, microbial genomics, necrotizing enterocolitis, neonates, iRep, metagenomics

## Abstract

During the first weeks of life, microbial colonization of the gut impacts human immune system maturation and other developmental processes. In premature infants, aberrant colonization has been implicated in the onset of necrotizing enterocolitis (NEC), a life-threatening intestinal disease. To study the premature infant gut colonization process, genome-resolved metagenomics was conducted on 343 fecal samples collected during the first 3 months of life from 35 premature infants housed in a neonatal intensive care unit, 14 of whom developed NEC, and metaproteomic measurements were made on 87 samples. Microbial community composition and proteomic profiles remained relatively stable on the time scale of a week, but the proteome was more variable. Although genetically similar organisms colonized many infants, most infants were colonized by distinct strains with metabolic profiles that could be distinguished using metaproteomics. Microbiome composition correlated with infant, antibiotics administration, and NEC diagnosis. Communities were found to cluster into seven primary types, and community type switched within infants, sometimes multiple times. Interestingly, some communities sampled from the same infant at subsequent time points clustered with those of other infants. In some cases, switches preceded onset of NEC; however, no species or community type could account for NEC across the majority of infants. In addition to a correlation of protein abundances with organism replication rates, we found that organism proteomes correlated with overall community composition. Thus, this genome-resolved proteomics study demonstrated that the contributions of individual organisms to microbiome development depend on microbial community context.

## IMPORTANCE

Humans are colonized by microbes at birth, a process that is important to health and development. However, much remains to be known about the fine-scale microbial dynamics that occur during the colonization period. We conducted a genome-resolved study of microbial community composition, replication rates, and proteomes during the first 3 months of life of both healthy and sick premature infants. Infants were found to be colonized by similar microbes, but each underwent a distinct colonization trajectory. Interestingly, related microbes colonizing different infants were found to have distinct proteomes, indicating that microbiome function is not only driven by which organisms are present, but also largely depends on microbial responses to the unique set of physiological conditions in the infant gut.

## INTRODUCTION

Infants have high levels of between-individual variation in microbiome composition compared with adult humans (1, 2). Variation in the infant microbiome exists at both the species and strain levels (3, 4). During the first 1 to 2 years of life, the gut microbiome of infants begins to converge upon an adult-like state (2, 5). However, aberrations in this process may contribute to diseases such as type 1 and 2 diabetes, irritable bowel disease, and necrotizing enterocolitis (NEC) in premature infants (6–11). Because establishment of the microbiome is a key driver of immune system development, changes in the process of colonization may have lifelong implications, even if they do not result in a drastically different microbiome composition later in life (12, 13).

Infants born prematurely have microbial communities that are lower in diversity than those of full-term infants and are susceptible to life-threatening diseases such as NEC (4, 14–17). While it has long been thought that bacterial infection may contribute to NEC pathogenesis, strain-resolved microbial community analysis has not identified a single pathogen that is responsible for the disease (3). However, it is still likely that microbial communities play an important role, with the context-dependent metabolism of specific strains potentially critical to infant health and disease. Recent studies have applied proteomics and metabolomics to premature infant gut microbiomes to measure functional profiles in healthy premature infants and in those that went on to develop NEC (18, 19). Those studies reported temporal variation in the infant proteome and identified metabolites associated with NEC. However, further study is required to better understand the range of functional and developmental patterns during the microbial colonization process.

To investigate microbial community assembly and how microbes modulate their metabolism and replication rate during colonization, we conducted a combined metagenomics and metaproteomics study of the microbiome of both healthy premature infants and infants that went on to develop NEC. Microbiome samples were collected during the first 3 months of life with the goal of measuring the physiological changes in the dominant and ubiquitous bacterial species. Genomes assembled from metagenomes enabled analysis of microbial community membership and tracking of both community composition and replication rates over time. The availability of genome sequences made it possible to map protein abundance measurements to bacterial species and strains. Microbial communities were clustered into distinct types in order to provide the context for proteomics analyses. Statistical analyses showed that, while the species-specific and strain-specific proteomic profiles correlated with overall community composition, the proteomes of members of the same species and strain were largely infant specific. These analyses also showed that bacterial proteome features are correlated with infant development, health status, and antibiotics administration.

## RESULTS

### Metagenome sequencing and genome binning.

In order to study the developing gut microbiome, stool samples were collected during the first 3 months of life from 35 infants born prematurely and housed in the neonatal intensive care unit at Magee-Womens Hospital at the University of Pittsburgh Medical Center. Two of the infants in the study cohort (N1_017 and N1_019) developed sepsis, and 14 infants developed necrotizing enterocolitis (NEC) (Table 1). To study the gut microbiome, we analyzed 1,149 GBp of DNA sequences generated by our laboratory (3, 4, 20). These sequences were from 343 metagenomes (average, 3.3 GBp sequencing per sample) (see Fig. S1 and File S1a in Data Set S1 in the supplemental material). Metagenomes were assembled into 6.79 GBp of scaffolds of ≥1 kbp, which represented 92% of all sequenced DNA.

10.1128/mBio.00441-18.1 FIG S1  Metagenome sequencing and metaproteomics conducted on microbiome samples collected from premature infants. (a and b) Frequency of sample collection for metagenomics (a) and metaproteomics (b) based on infant day of life (DOL). (c and d) Metagenome sequencing data (c) and the percentage of each metagenome represented by assembled genome sequences ≥50% complete with ≤5% contamination (d). (e) The number of proteomics spectral counts that could be uniquely assigned to human or bacterial proteins. (f) The percentage of predicted proteins that could be detected in each sample. (g) The percentage of species-specific proteomes that could be detected for species where ≥10% of the proteome could be detected in at least one sample. (h) Histogram showing the distribution of the maximum percentages of the proteome detected for all species present in each sample. Download FIG S1, PDF file, 0.3 MB. Copyright © 2018 Brown et al. 2018 Brown et al. This content is distributed under the terms of the Creative Commons Attribution 4.0 International license.

10.1128/mBio.00441-18.10 DATA SET S1 Supplemental data files. Download DATA SET S1, XLSX file, 17.2 MB. Copyright © 2018 Brown et al. 2018 Brown et al. This content is distributed under the terms of the Creative Commons Attribution 4.0 International license.

**TABLE 1:** Infant medical information^a^

Infant	Study	Sex	Delivery	Mult. gest.	Gestational age (wks)	Birth wt (g)	Feeding	Condition	NEC diagnosis (DOL)
N1_003	NIH1	F	C-section	Single	26	822	Breast	Control	
N1_004	NIH1	F	C-section	N1_005	32	1,450	Formula	Control	
N1_008	NIH1	F	Vaginal	Single	32	1,230	Formula	NEC	9
N1_009	NIH1	M	C-section	Single	29	1,820	Combination	Control	
N1_011	NIH1	M	C-section	N1_012	26	523	Combination	NEC	34, 62
N1_014	NIH1	M	Vaginal	Single	32	2,035	Combination	Control	
N1_017	NIH1	F	Vaginal	Single	26	748	Combination	NEC	11
N1_018	NIH1	M	C-section	Single	29	1,133	Combination	Control	
N1_019	NIH1	F	C-section	N1_020, N1_021	24	731	Combination	Control	
N1_021	NIH1	F	C-section	N1_019, N1_020	24	697	Breast	NEC	32
N1_023	NIH1	F	Vaginal	Single	27	875	Breast	Control	
N2_031	NIH2	M	C-section	Single	26	773	Formula	Control	
N2_035	NIH2	M	Vaginal	Single	25	795	Breast	Control	
N2_038	NIH2	F	C-section	N2_039	30	1,381	Combination	Control	
N2_039	NIH2	F	C-section	N2_038	30	1,470	Combination	NEC	24
N2_060	NIH2	M	C-section	Single	30	1,878	Combination	Control	
N2_061	NIH2	M	Vaginal	Single	28	1,184	Combination	NEC	9, 34
N2_064	NIH2	M	Vaginal	Single	28	1,100	Combination	Control	
N2_065	NIH2	F	Vaginal	Single	25	841	Combination	Control	
N2_066	NIH2	F	Vaginal	Single	28	1,028	Breast	Control	
N2_069	NIH2	M	C-section	N2_070	26	637	Breast	NEC	32
N2_070	NIH2	F	C-section	N2_069	26	633	Combination	Control	
N2_071	NIH2	M	C-section	Single	25	754	Combination	NEC	31
N2_088	NIH2	F	C-section	N2_089	28	1,057	Formula	Control	
N2_093	NIH2	M	C-section	Single	26	924	Breast	NEC	12
N3_172	NIH3	M	C-section	Single	28	1,250	Breast	NEC	37, 54
N3_173	NIH3	M	C-section	Single	29	1,530	Breast	NEC	25
N3_174	NIH3	F	C-section	Single	30	980	Breast	Control	
N3_175	NIH3	M	Vaginal	Single	29	1,480	Combination	Control	
N3_176	NIH3	M	C-section	Single	28	990	Combination	Control	
N3_177	NIH3	F	Vaginal	Single	28	900	Combination	Control	
N3_178	NIH3	M	Vaginal	Single	32	2,050	Combination	NEC	16
N3_182	NIH3	M	C-section	Single	39	3,010	Combination	NEC	6
N3_183	NIH3	M	Vaginal	Single	32	2,410	Combination	NEC	11
S2_010	NIH3	M	C-section	Single	32	1,810	Combination	Control	

a Mult. gest., multiple gestations; F, female; M, male.

Scaffolds assembled from metagenomes were grouped into 3,643 bins, 1,457 of which were ≥50% complete with ≤5% contamination (Fig. S2; see also File S2 at https://doi.org/10.1101/217950). These genomes were assigned to 270 groups approximating different bacterial subspecies based on sharing ≥98% average nucleotide identity (ANI) (see File S1b in Data Set S1). These genomes accounted for 91% of the total sequencing. Genomes suitable for index of replication (iRep) rate analysis (≥75% complete with ≤175 fragments/Mbp and ≤5% contamination) were available for 193 genome clusters (21).

10.1128/mBio.00441-18.2 FIG S2  ESOM genome binning. Genome binning was conducted based on emergent self-organizing map (ESOM) clustering of scaffolds assembled from individual metagenomes. Data points represent 3-kbp fragments of assembled scaffolds. Coloring is based on the species-level assignment of reconstructed genomes. The map is periodic, and red boxes indicate a single period. Download FIG S2, PDF file, 38.2 MB. Copyright © 2018 Brown et al. 2018 Brown et al. This content is distributed under the terms of the Creative Commons Attribution 4.0 International license.

### Protein quantification by metaproteomics.

Across all metagenomes, 5,233,047 proteins were predicted, 897,520 of which were from a nonredundant set of representative genomes clustered at 98% ANI. Proteins clustered into 121,746 putative families (see File S3 at https://doi.org/10.1101/217950). Metaproteomics measurements were conducted on 87 metagenome-matched samples that spanned 16 infants, 6 of whom developed NEC and 1 of whom (N1_019) was diagnosed with sepsis (Fig. S1). Conducting metagenomics and metaproteomics on the same samples was critical for obtaining an appropriate database for matching peptides to proteins. On average, 71,676 unique bacterial spectral counts were detected per sample, with an average of 33% of predicted bacterial proteins identified (Fig. S1; also see File S1b in Data Set S1 and File S4 at https://doi.org/10.1101/217950).

### Premature infants were colonized by genetically similar organisms, and microbial communities clustered into seven primary types.

**FIG 1:**
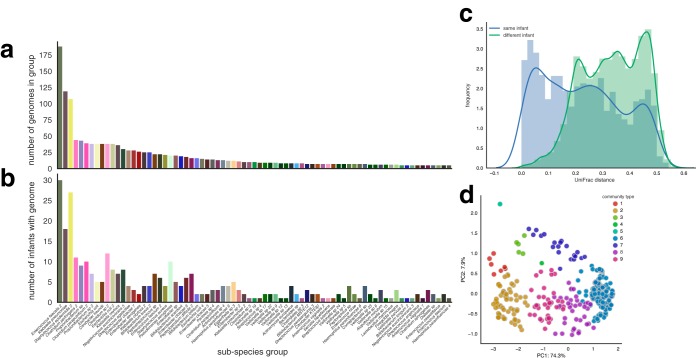
Premature infant gut microbial communities associated into seven primary types. Genomes reconstructed from metagenomes were clustered into subspecies groups based on sharing 98% average nucleotide identity (ANI). (a) The number of genomes assigned to each group. (b) The number of infants with a reconstructed genome from the group. Groups composed of five or more genomes are shown. (c) Pairwise weighted UniFrac distances calculated between all microbiome samples based on genome sequence ANI and abundance. (d) Principal-coordinate analysis (PCoA) clustering of samples based on weighted UniFrac distances. Samples are colored based on community type assignment.

The majority of infants were colonized by Enterococcus faecalis, Klebsiella pneumoniae, and Staphylococcus epidermidis (Fig. 1a and b). Overall, infants that developed NEC were colonized by organisms genetically similar to those colonizing other infants, and most genotypes were seen in only one infant. No individual species was strongly associated with NEC (Fig. S3).

10.1128/mBio.00441-18.3 FIG S3  Infants that developed NEC and healthy controls were colonized by genetically similar bacteria. Presence (dark boxes) and absence (white boxes) of members of bacterial subspecies in microbial communities from different infants. Subspecies were identified based on sharing ≥98% genome average nucleotide identity (ANI) and were determined to be present if ≥97% of the genome was covered by an average of ≥2 reads. Hierarchical clustering was conducted based on unweighted UniFrac distances calculated between infant genome inventories. Download FIG S3, PDF file, 0.2 MB. Copyright © 2018 Brown et al. 2018 Brown et al. This content is distributed under the terms of the Creative Commons Attribution 4.0 International license.

**FIG 2:**
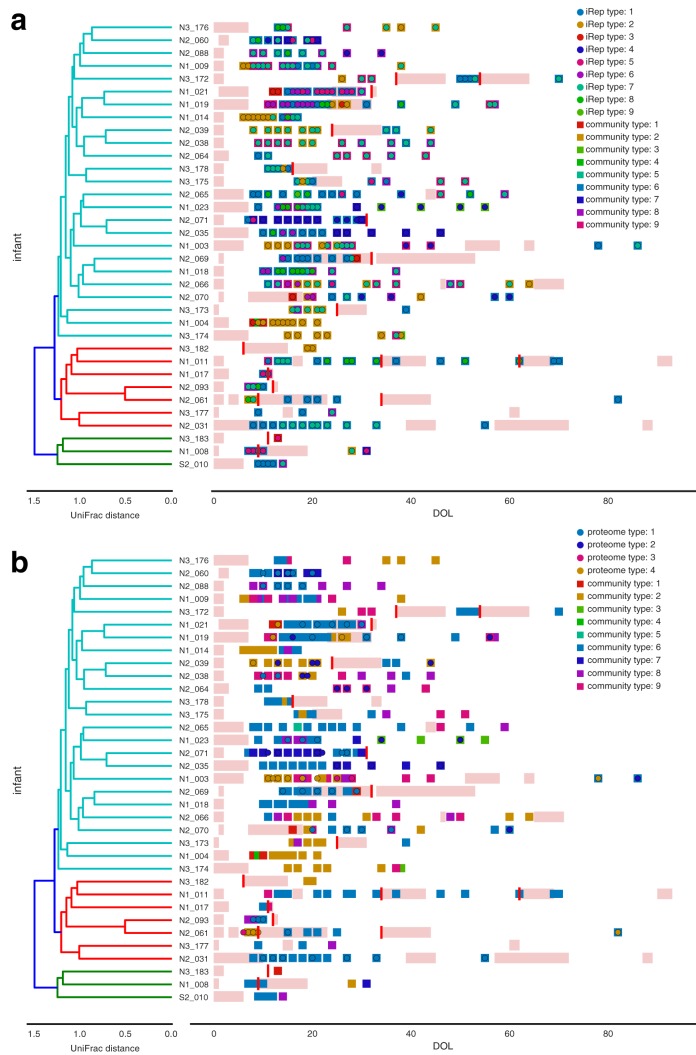
Microbial colonization patterns for preterm infants. Samples were clustered into types based on microbial community composition (“community type”), bacterial iRep profiles (“iRep type”), and overall bacterial proteome composition (“proteome type”). The microbial community type is shown along with iRep (a) and proteome (b) types. Infants are arranged based on hierarchical clustering of unweighted UniFrac distances calculated based on the set of genomes recovered from each infant (Fig. S3). Antibiotics administration data are indicated with pink bars and NEC diagnosis data with red bars. DOL, days of life.

The premature infant microbiome was found to be highly variable. In some cases, samples collected from an infant at subsequent time points were as different from earlier samples as those collected from other infants (Fig. 1c). Communities were clustered based on species membership and abundance in order to identify microbial consortia common during the colonization process. In order to account for both genomic differences and organism abundance, clustering was conducted based on weighted UniFrac distances, where the tree used for calculating UniFrac was constructed using genome ANI. Nine distinct community types were identified, seven of which were composed of samples collected from multiple infants and were thus considered primary types (Fig. 1d; see also Fig. S4 and S5). Each community type was characterized by the dominance of different community members (Fig. S6). Microbiomes from different infants clustered into the same community type, and the microbiome of individual infants was found to switch types, sometimes multiple times, during the colonization process (Fig. 2). Although infants shared community types, overall colonization patterns were not replicated across infants. Microbiomes associated with infants that did and did not go on to develop NEC were often classified in the same community type. In some cases, switches preceded onset of NEC, but no type or switch could explain all cases of NEC.

10.1128/mBio.00441-18.4 FIG S4  Studied infant gut microbial communities associate into seven primary community types. (a) Hierarchical clustering was conducted based on the abundance of bacterial subspecies using weighted UniFrac distances. Microbial community types are identified by colored boxes. Metadata are shown for each sample and are indicated with an asterisk if significantly correlated with microbial community abundance data (PERMANOVA or Mantel test *P* value ≤ 0.01). (b to i) PCoA clustering of microbial communities with associated metadata: antibiotics administration (b), infant (c), developmental age (d; number of days since conception: gestational age + day of life [Ga+ DOL]), proteome type (e), iRep type (f), infant health (g), days prior to NEC diagnosis (h; DOL − NEC diagnosis), and human proteome type (i). Download FIG S4, PDF file, 5.1 MB. Copyright © 2018 Brown et al. 2018 Brown et al. This content is distributed under the terms of the Creative Commons Attribution 4.0 International license.

10.1128/mBio.00441-18.5 FIG S5  Microbial community abundance and replication rate profiles. Relative abundance (bars) and iRep replication rate (scatter plot) values for bacterial subspecies colonizing studied premature infants are indicated. The 5 days following antibiotics administration are indicated with a color gradient. Download FIG S5, PDF file, 2.4 MB. Copyright © 2018 Brown et al. 2018 Brown et al. This content is distributed under the terms of the Creative Commons Attribution 4.0 International license.

10.1128/mBio.00441-18.6 FIG S6  Microbial community types are distinguished by their abundant members. Rank abundance curves showing the average and range (95% confidence interval) of relative abundance values for the subspecies groups associated with each community type are presented. Download FIG S6, PDF file, 0.1 MB. Copyright © 2018 Brown et al. 2018 Brown et al. This content is distributed under the terms of the Creative Commons Attribution 4.0 International license.

### Microbial replication rates and proteins.

iRep is a newly developed method that enables measurement of bacterial replication rates based on metagenome sequencing data when high-quality draft genome sequences are available (21). We applied the iRep method using genomes recovered from metagenomes sequenced for each infant in the study and quantified 1,328 iRep replication rates from 330 samples. Sample clustering was conducted based on community iRep profiles, identifying nine distinct iRep types that were correlated with community type (Mantel test *P* value of 1 × 10^−3^) (Fig. 2a). Likewise, analysis of protein family abundance clustered samples into four distinct proteome types, which also correlated with community type (Mantel test *P* value of 1 × 10^−3^) (Fig. 2b). Interestingly, there were several cases in which the iRep and/or proteome type switched when the community type was constant or in which the community type switched but the iRep and/or proteome type remained constant.

### Microbiome development.

**FIG 3:**
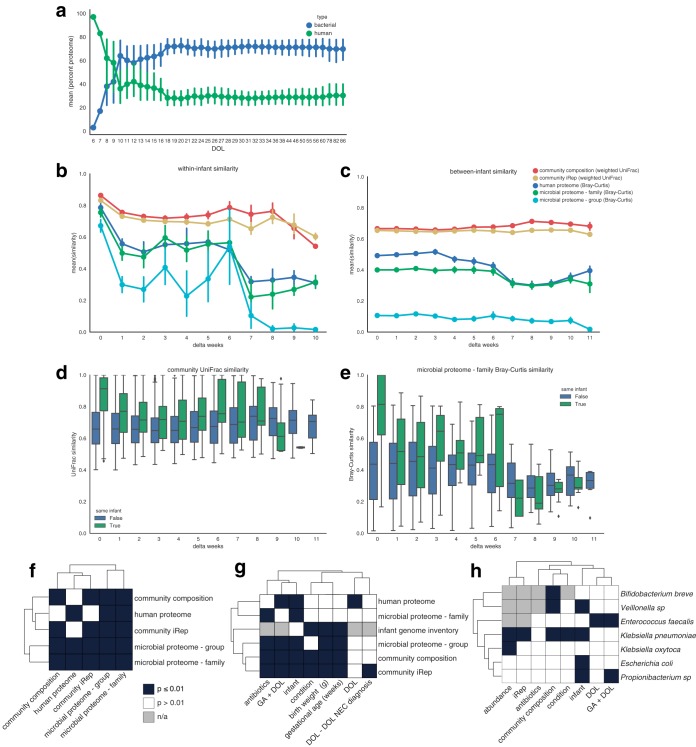
Microbiome stability and correlations. (a) The relative contributions of human and bacterial proteins to overall proteome composition during development of the premature infant gut. (b and c) Similarity measurements for microbiomes sampled either from the same infant (b) or from different infants (c). (d and e) Comparison of similarity measurements calculated for samples collected either from the same infant or from different infants based either on weighted microbial community UniFrac (d) or weighted microbial proteome Bray Curtis (e) measurements. (f to h) Correlations calculated with human proteome and microbial community data (f) or with infant metadata (g) and determined based on microbial species (h). Correlations were calculated using PERMANOVA or Mantel tests. family, protein family analysis; group, analysis of proteins clustered at 97% amino acid identity.

Peptide spectral counts were matched to infant-specific databases containing both human and microbial proteins. This allowed the relative proportions of human and microbial proteins to be determined for each time point. Samples were dominated by human proteins during the first 10 days of life (DOL), and then microbial proteins became dominant around DOL 18. Ratios of human versus bacterial protein abundances show that the premature infant gut microbiome is established over a period of approximately 2 weeks (Fig. 3a).

The presence of multiple data types (microbial community abundance and iRep, microbial community proteome composition, and human proteome composition) enabled tracking of various aspects of human and microbiome development during the first months of life (Fig. 3b and c). All measurements from an infant were stable within the time scale of a week but diverged over time. Interestingly, communities from different infants neither converged nor diverged over time in terms of similarity based on three of these five metrics. However, we observed that human proteome measurements and microbial protein family abundances from different infants became increasingly different when samples with time separations of greater than 3 weeks were compared. Overall, the microbial proteome was more variable (higher variance) than the community composition (Fig. 3d and e). After approximately 2 weeks, the microbial community abundance data and proteome measurements collected from the same infant became as different from each other as from those of samples collected from other infants.

The majority of human and microbiome features recorded in our analyses were correlated with one another (Fig. 3f). However, an exception was that microbial community abundance and iRep were not correlated with human proteome composition (Mantel test *P* value > 0.01). This is interesting in that it shows that there is no strong connection between the overall human proteome and either the composition or replication activity of the microbiome.

As shown in Fig. 3g, microbial features were also correlated with a variety of infant factors, including infant health and development (gestational age [Ga] and weight), as well as with antibiotics administration (Mantel test or permutational multivariate analysis of variance [PERMANOVA] *P* value ≤ 0.01). Notably, whether or not an infant developed NEC (condition) correlated with several microbiome factors (infant genome inventory and both community composition and iRep) but not with proteome measurements. However, these correlations were in part driven by antibiotics, as only iRep was correlated with infant condition when excluding samples collected during or within 5 days of antibiotics administration. Regardless of the influence of antibiotics on the microbiome, microbial responses to treatment likely impact infant health.

### Different species expressed various amounts of their proteome in the infant gut.

**FIG 4:**
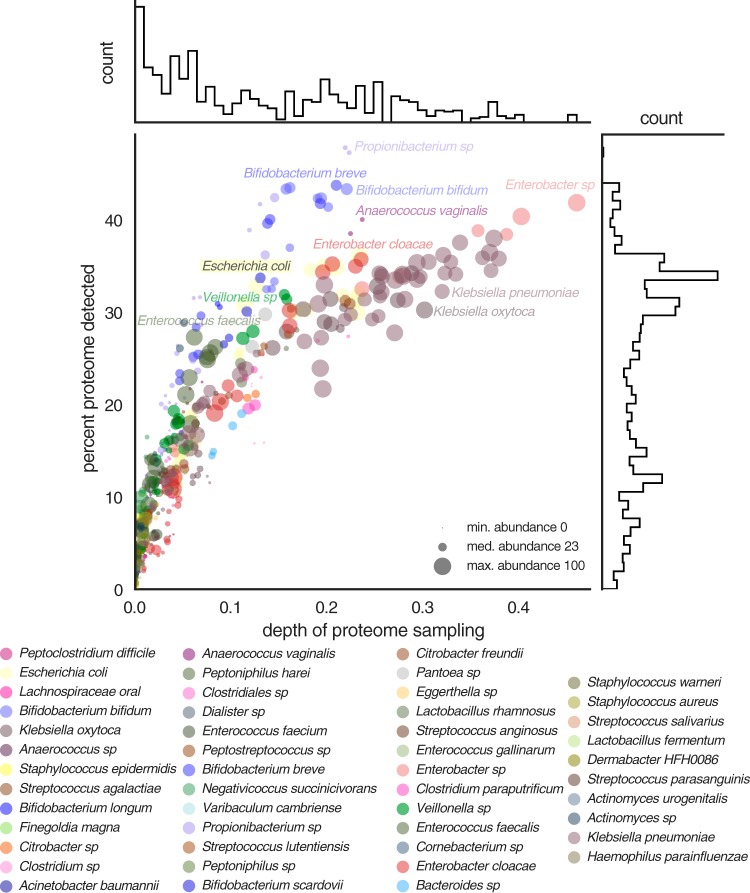
Proteome detection for species colonizing premature infants. Depths of proteome sampling for organisms in each sample are compared to percentages of predicted proteins that could be detected. Data point sizes and histograms are scaled based on organism abundance as determined by metagenome sequencing.

Microbes present in the gut environment are not expected to express their complete complement of proteins at all times. In order to investigate the extent of proteome expression for different bacteria, we compared the depth of proteome sampling for each organism to the percentage of the predicted proteome that could be detected (Fig. 4). The median level of proteome detection across all samples was 11%, but this was largely due to low sampling depth. Higher depth of proteome sampling corresponded with detection of a larger fraction of the predicted proteins. The median percentage of the proteome detected for organisms with the best detection in each sample was 31% (maximum [max.], 48%). For several frequently detected colonists, including Klebsiella pneumoniae, Klebsiella oxytoca, and members of the genus *Enterobacter*, maximum proteome expression was ~50%. However, *Propionibacterium* sp., Anaerococcus vaginalis, and members of the genus *Bifidobacterium* expressed a greater proportion of their genes than other organisms. We infer that these bacteria may be specifically adapted to environments and resource availability within the infant gut, whereas other bacteria may maintain capacities that enable adaption to other environments.

### Members of the same bacterial species replicated at different rates during colonization.

**FIG 5:**
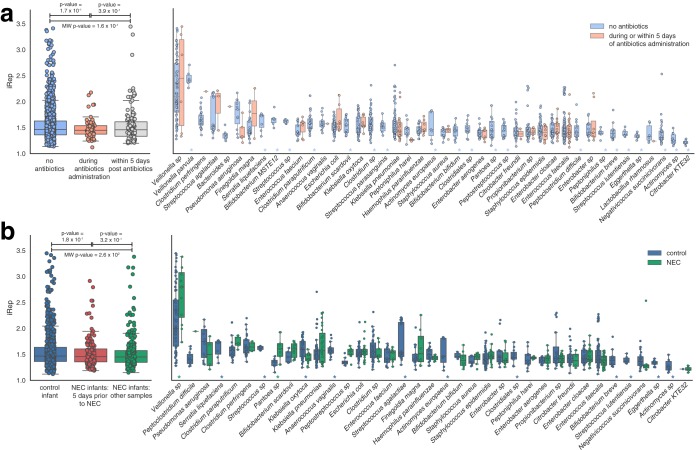
Replication rates for bacteria colonizing premature infants. (a) Replication rates for bacteria sampled during periods with or without antibiotics administration. (b) Replication rates associated with infants that did and did not go on to develop NEC. Statistically significant differences between replication rates observed for individual species under different conditions are indicated with an asterisk (MW *P* value, ≤0.01). Data represent all species with at least five observations.

Across all infants, Streptococcus agalactiae, Pseudomonas aeruginosa, Klebsiella pneumoniae, and members of the genera *Veillonella* and *Clostridium* exhibited some of the highest replication rates (see File S1c in Data Set S1). iRep values for organisms sampled in this cohort during or immediately after antibiotics administration were not significantly different from those determined at other time points (Fig. 5a). This indicates that populations present after antibiotics administration were both resistant to antibiotics and continuing to replicate. Members of several species (*Veillonella* sp., Streptococcus agalactiae, Finegoldia magna, and others) were replicating quickly during or immediately following antibiotic treatment. However, we did not detect iRep values that were higher overall following antibiotics administration, although this was reported previously (21). Most species were found to be replicating only in the absence of antibiotics, consistent with their susceptibility to the treatment.

### Species-specific proteomic profiles are associated with infant and microbiome features.

Relative protein abundance levels were determined for each genome and tracked across samples. This identified population-specific proteome profiles and enabled us to test for correlations with various human and microbial properties (Fig. 3h; see also Fig. S1 and File S1d in Data Set S1). *Veillonella* spp., Klebsiella pneumoniae, Escherichia coli, and *Propionibacterium* sp. all had infant-specific profiles (PERMANOVA *P* value ≤ 0.01), indicating that although similar organisms were colonizing different infants, each population was expressing a different complement of proteins. The K. pneumoniae and *Veillonella* proteomes also correlated with community type, as did the Bifidobacterium breve proteome (Mantel test *P* value ≤ 0.01), showing that the populations responded to overall microbial community context. Interestingly, both Enterococcus faecalis and *Propionibacterium* sp. exhibited proteomes that were also correlated with infant development, and the K. pneumoniae proteome correlated with both iRep and infant health. Although overall microbial proteome correlated with antibiotics administration, species-specific proteome profiles did not; however, this may have been due to a lack of available data for the same species in multiple samples with and without antibiotics.

Because of the existence of 35 samples in which ≥10% of the K. pneumoniae proteome could be detected (max. = 38%; median = 25%), correlations between individual protein abundances and iRep could be determined. Among the proteins positively correlated with iRep were a transcriptional regulator (LysR), proteins involved in cell wall biogenesis, and ribosomal proteins (Pearson value, ≥0.5; false-discovery-rate [*q*] value ≤ 0.01; observed in ≥15 samples) (see File S1e in Data Set S1).

### Infants were colonized by different strains with distinct proteomes.

**FIG 6:**
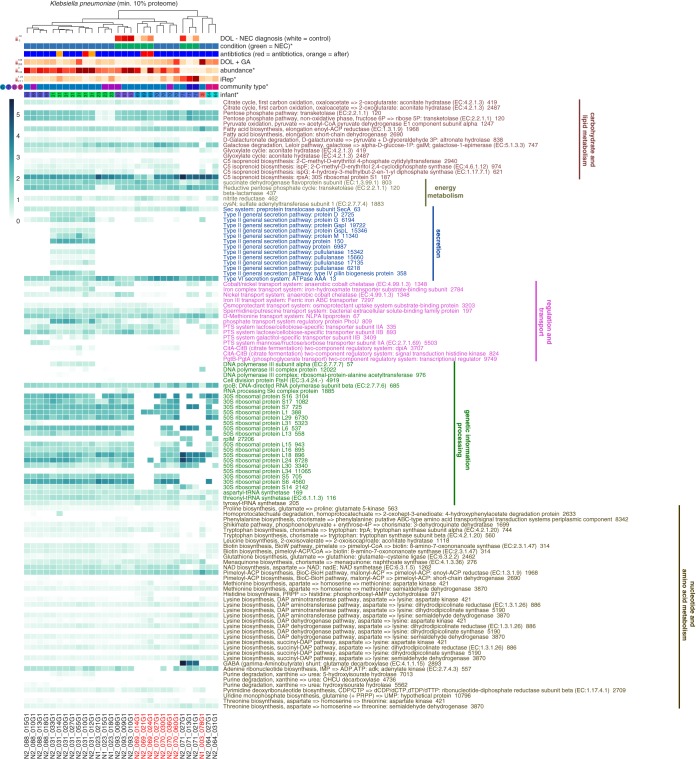
Klebsiella pneumoniae proteins with infant-specific abundance profiles. Hierarchical clustering was conducted on all K. pneumoniae protein families, showing that the strains colonizing different infants had distinct proteomic profiles. Infant and species metadata are shown for each sample. Metadata significantly correlated with the K. pneumoniae proteome are indicated with an asterisk (PERMANOVA or Mantel test *P* value ≤ 0.01). Protein families that correlated with at least one infant are shown in the heat map (edgeR *q* value ≤ 0.01). Samples colonized by the same K. pneumoniae strain are shown with red text.

The finding that K. pneumoniae, E. coli, *Propionibacterium* sp., and *Veillonella* spp. have infant-specific proteomes raised the issue of whether or not each infant was being colonized by different strains. Genome sequences that were ≥50% complete with ≤5% contamination that were assembled for each species from each infant were compared with one another, and hierarchical clustering conducted on pairwise ANI values was used to delineate strains (Fig. S7). Clustering showed that in most cases each infant was indeed colonized by distinct strains, which proteomics analysis showed were functionally distinct. However, there were a few notable exceptions. Twin infants N2_069 and N2_070, as well as infant N1_003, were colonized by the same strain of K. pneumoniae. The proteomic profiles for the strains colonizing N2_069 and N2_070 were more similar to one another than they were to profiles recovered from other strains; however, they were still distinguishable (Fig. 6). Likewise, the same strain of *Propionibacterium* sp. colonized twin infants N2_038 and N2_039. As with shared strains of K. pneumoniae, their functional profiles clustered together but were still distinguishable from one another (Fig. S8).

10.1128/mBio.00441-18.7 FIG S7  Hierarchical clustering of genomes for members of the same subspecies group. dRep results show ANI clustering of assembled genomes. Genome names indicate the metagenome that each genome was assembled from the data, see File S1b in Data Set S1. Clustering dendrograms show that most infants were colonized by different strains. Download FIG S7, PDF file, 5.7 MB. Copyright © 2018 Brown et al. 2018 Brown et al. This content is distributed under the terms of the Creative Commons Attribution 4.0 International license.

10.1128/mBio.00441-18.8 FIG S8  Multiple species have infant-specific proteome profiles. (a) Analysis of *Veillonella* sp. genomes shows the presence of four different species. (b to e) Proteome profiles for different species colonizing premature infants. Hierarchical clustering was conducted based on all detected protein families, and the data show that the strains colonizing different infants typically have distinct proteomic profiles. Infant and species metadata are shown for each sample. Metadata significantly correlated with the species proteome are indicated with an asterisk (PERMANOVA or Mantel test *P* value ≤ 0.01). Protein families that correlated with at least one infant are shown in the heat map (edgeR *q* value ≤ 0.01). Samples colonized by the same strain are shown with colored text. Download FIG S8, PDF file, 0.1 MB. Copyright © 2018 Brown et al. 2018 Brown et al. This content is distributed under the terms of the Creative Commons Attribution 4.0 International license.

Analysis showed that only a few proteins were responsible for distinguishing proteomes of the same bacterial types in different infants (Fig. 6; see also Fig. S8 and File S1d in Data Set S1). Common among these were proteins involved in nucleotide, amino acid, carbohydrate, and lipid metabolism. Also notable were several proteins produced by K. pneumoniae that were involved in central carbohydrate metabolism and in both galactose degradation and d-galacturonate degradation, indicating different carbon preferences for strains colonizing different infants (Fig. 6). Proteins involved in bacterial secretion were differentially abundant between K. pneumoniae strains colonizing different infants, indicating variations in secretion potential that could affect human-microbe interactions. Relatedly, the abundances of proteins involved in transport of metals, ions, citrate, and several sugars also differed between infants.

### Low microbiome diversity was associated with both antibiotics administration and NEC.

**FIG 7:**
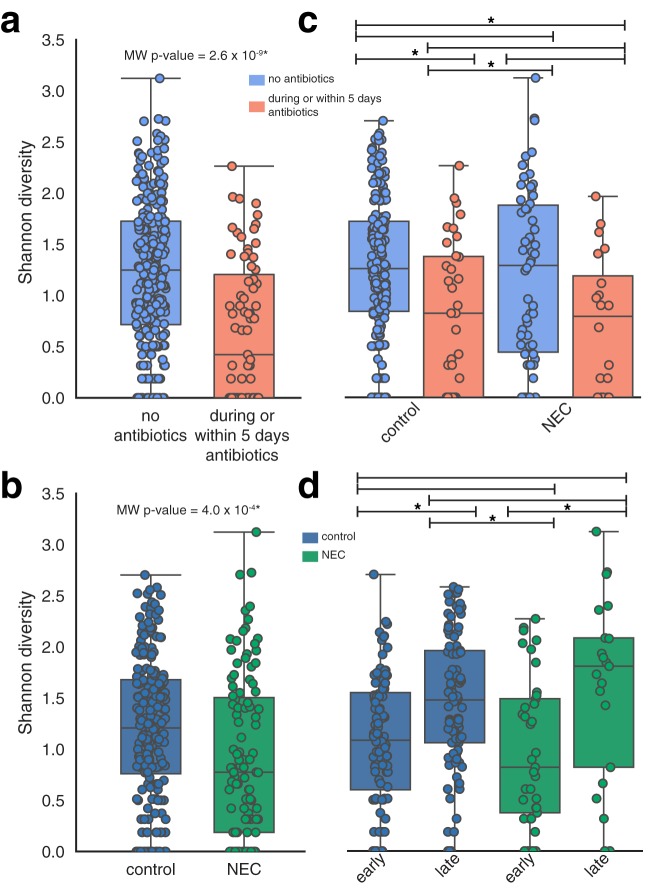
Microbial community diversity. (a) Shannon diversity measurements for microbial communities associated with infants during periods with or without antibiotics administration. (b to d) Shannon diversity data resulting from comparisons between infants that did and did not go on to develop NEC. Significant differences are indicated with an asterisk (MW *P* value ≤ 0.01). “Early” samples were collected prior to Ga+ DOL 220. Samples collected after NEC diagnosis were excluded from the calculations whose results are shown in panels c and d.

Microbiome diversity was lower during or within 5 days of antibiotics administration than at other time points (Mann-Whitney [MW] *U* test; MW *P* value, 2.6 × 10^−9^; Fig. 7a), and the microbiome of infants that developed NEC was typically less diverse than that of healthy infants (MW *P* value, 4 × 10^−4^; Fig. 7b). However, the difference in diversity between healthy and NEC infants was driven by the fact that NEC infants more frequently receive antibiotics (Fig. 2). Comparing data corresponding to the periods with and without antibiotics, the levels of microbiome diversity for healthy and NEC infants (pre-NEC diagnosis) were indistinguishable (Fig. 7c). Excluding antibiotics samples, both groups of infants had higher diversity microbial communities later in development (post-gestational age-corrected day of life [post-Ga+ DOL] 220; Fig. 7d).

### Microbial community composition was correlated with infant health.

Premature infants that developed NEC had different microbial community abundance profiles (PERMANOVA *P* value of 3 × 10^−3^; Fig. S4g). Interestingly, there were a variety of species that were detected in healthy infants but never detected in those that developed NEC; however, the opposite was not true. It should be noted that species unique to healthy infants were not consistently detected. No species identified 5 days prior to NEC diagnosis showed a significant difference in abundance or was unique to NEC infants.

Overall community composition was also correlated with each infant, antibiotics administration, birth weight, gestational age, and gestational age corrected day of life (Ga+ DOL; PERMANOVA or Mantel test *P* value ≤ 0.01; Fig. S4). Several species, including *Enterobacter* sp., *Propionibacterium* sp., and *Peptostreptococcus* sp., represented more-abundant members of communities associated with infants that developed NEC (*q* value ≤ 0.01 after excluding samples collected within 5 days of antibiotics administration) (see File S1f in Data Set S1). *Vellonella* sp. replicated faster in NEC infants, while several groups of organisms were replicating faster in control infants, including members of the genera *Anaerococcus*, *Klebsiella*, *Actinomyces*, *Eggerthella*, *Streptococcus*, *Clostridiales*, and *Bifidobacterium* (MW *P* value ≤ 0.01 [excluding samples collected within 5 days of antibiotics administration]) (see File S1c in Data Set S1). Several different species were active in control infants but were not detected in the infants that went on to develop NEC. However, the combined iRep values collected from infants that did and did not go on to develop NEC were not significantly different, even considering only samples collected within the 5 days prior to NEC diagnosis (Fig. 5b).

### Microbial proteins associated with proteome type, antibiotics administration, and NEC.

As described above, we used protein abundance patterns to cluster microbial community proteomes into functionally distinct proteome types. Statistical analysis identified 3,085 differentially abundant proteins distinguishing proteome types (edgeR false-discovery-rate [*q*] value ≤ 0.01) (see File S1g in Data Set S1). Of these, 461 were found to distinguish only one proteome type from all others. Notable among all of these proteins were those involved in central carbohydrate metabolism and energy metabolism (Fig. S9). Proteome types differ in terms of the amount and variety of carbon degradation enzymes as well as in the propensity for aerobic versus anaerobic respiration (based on the abundance of oxidases and reductases).

10.1128/mBio.00441-18.9 FIG S9  Proteome types are distinguished by the abundance of proteins from different KEGG modules. Hierarchical clustering of proteome types was conducted based on the abundance of proteins associated with each module. The relative abundances of proteins associated with each module were summed for each sample, and then the average was taken across all samples associated with each proteome type. Download FIG S9, PDF file, 0.1 MB. Copyright © 2018 Brown et al. 2018 Brown et al. This content is distributed under the terms of the Creative Commons Attribution 4.0 International license.

Samples collected during antibiotics treatment were enriched in 56 different proteins (identified in two or more treated infants; edgeR *q* value, ≤0.01) (see File S1g in Data Set S1). Among these proteins were those involved in secretion, transcription, and DNA degradation. Along with iRep results, the findings indicate that a subset of organisms remained active in the presence of antibiotics.

Although overall community proteome abundance profiles were not correlated with NEC, microbial proteins from 160 different protein families, many with no known function, were more abundant in samples from infants that went on to develop NEC (identified in two or more NEC infants; edgeR *q* value ≤ 0.01) (see File S1g in Data Set S1). Annotated proteins were dominantly involved in transport of ions, metals, and other substrates; iron acquisition; and both motility and chemotaxis. Among the proteins responsible for iron scavenging was subunit E of enterobactin synthase, a high-affinity siderophore involved in iron acquisition, which is often used by pathogenic organisms. Also more abundant was outer membrane receptor FepA, which is involved in transporting iron bound by extracellular enterobactin. Subunit F of enterobactin synthase was also identified in NEC infants, as were an iron-enterobactin ABC transporter substrate-binding protein and an enterobactin esterase. The abundance of this protein suggests a possible role for iron acquisition by organisms that may contribute to disease onset. Interestingly, 21 K. pneumoniae proteins were correlated with NEC, including a ferrous iron transporter (family 2834) that was 3.9-fold more abundant in two infants that developed NEC. The abundance of this protein was also correlated with infant, proteome type, community type, and antibiotics administration.

## DISCUSSION

Most studies to date have focused on the composition of the gut microbiome, typically at the low resolution afforded by 16S rRNA gene amplicon methods. We used genome-resolved time-series metagenomics in conjunction with iRep replication rate and metaproteomics measurements to obtain a more comprehensive view of the colonization process. The data set included information about the gut colonization trajectories of both healthy infants and infants that went on to develop NEC, enabling exploration of microbiome variability, at both the community composition and organism functional levels.

Microbial communities were classified into types based on the mixture of organisms present. Interestingly, most types occurred in multiple infants, a result that indicates the tendency of gut-colonizing bacteria to form networks of interaction, possibly based on metabolic complementarity. An important factor determining the community type present may be the specific organisms that are introduced and the extent to which they are able to colonize. Other factors that may dictate the community type include human genetic selection, diet, and antibiotics administration. Within a single infant, community types often switched several times over the observation period. Given the lack of evidence for consistent transitions from one type to another across multiple infants, the high degree of variation in iRep replication rates observed for members of the same species, and a lack of convergence of communities in different infants, we conclude that colonization is a chaotic process.

Overall microbial physiology, as measured by whole proteome abundance patterns, was more dynamic than community composition. Thus, metagenomics-enabled proteomic analyses indicate that functional flexibility does not depend on addition or loss of organisms. Shifts in the importance of specific pathways or metabolisms with environmental conditions would not be apparent in studies that use only organism identification or metabolic potential predictions.

It is possible that the onset of NEC is due to high growth rates of potential pathogens within communities that are imbalanced due to low species richness, ultimately resulting in overgrowth by a pathogen. For this reason, we compared levels of microbial community diversity and composition, growth rates, and metabolic features in infants that did and did not develop NEC. A clear finding of this study that was evident from prior research (17) was that microbial communities associated with infants that develop NEC are of lower diversity than those of control infants. However, this was due to the frequency of antibiotics administration for NEC infants. Regardless of the cause of the lower-diversity communities, microbial activities throughout the colonization process, including during periods of antibiotics administration, are likely important to infant health.

Several different species had higher relative abundance in infants that developed NEC, but none of these species were consistently associated with the disease. The correlation could have been the consequence of the loss of other organisms from the community rather than of their higher absolute abundance. Interestingly, *Veillonella* spp. were found to replicate more quickly in infants with NEC than in control infants. This may be medically important, but additional examples are needed to establish a link between rapid growth and NEC.

Surprisingly, whether or not an infant developed NEC was not correlated with overall proteome composition. However, there were specific proteins that were associated with NEC, notably several involved in iron scavenging. Given that this is an important process often associated with pathogenesis, it is possible that increased activity of iron-scavenging pathways could contribute to organism proliferation and onset of NEC. In addition, the Klebsiella pneumoniae proteome, including a protein involved in transport of iron, was correlated with NEC. This is intriguing, considering the prior finding that supplementation of lactoferrin, an abundant breast milk protein involved in modulating iron levels in the gut, decreases the risk of developing necrotizing enterocolitis (22, 23). Overall, these findings indicate that fine-scale, species-specific proteins are important for understanding disease onset. Although the microbial community and specific microbial proteins were correlated with NEC, no individual organism or protein was significantly more abundant in all cases. This finding supports the hypothesis that NEC is a multifaceted disease with multiple routes that lead to onset.

Although species-specific proteome profiles were correlated with community composition, they were largely infant specific. This is an interesting observation because it implies feedback between human physiological conditions in the gut, which likely vary substantially from infant to infant and over time, and microbiome function.

## MATERIALS AND METHODS

### Sample collection and metagenome sequencing.

Samples were collected, processed for metagenome sequencing, and sequenced as part of three prior studies (accession numbers are provided in File S1a in Data Set S1 in the supplemental material) (3, 4, 20). Stool samples were collected from infants and stored at −80°C. DNA was extracted from frozen fecal samples using a Mo Bio PowerSoil DNA isolation kit, with modifications (4). DNA libraries were sequenced on an Illumina HiSeq instrument for 100 or 150 cycles (Illumina, San Diego, CA). All samples were collected with parental consent.

### Metagenome assembly and genome binning.

We reassembled and analyzed metagenomes generated as part of a prior study, referred to as NIH1 (4). The data were processed in a manner consistent with the two other prior studies analyzed, referred to as NIH2 (20) and NIH3 (3). All raw sequencing reads were trimmed using Sickle (https://github.com/najoshi/sickle). Each metagenome was assembled separately using IDBA_UD (24). Open reading frames (ORFs) were predicted using Prodigal (25) with the option to run in metagenome mode. Predicted protein sequences were annotated based on USEARCH (–ublast) (26, 27) searches against UniProt (28), UniRef100 (29), and KEGG (30, 31). Scaffold coverage was calculated by mapping reads to the assembly using Bowtie2 (32) with default parameters for paired reads.

Scaffolds from NIH1 infants were binned to genomes using emergent self-organizing maps (ESOMs) generated based on time-series abundance profiles (15, 33). Reads from every sample were independently mapped to every assembly using SNAP (34), and the resulting coverage data were combined. Coverage was calculated over nonoverlapping 3 KBp windows. Coverage values were normalized by sample first, and then the values for each scaffold fragment were normalized from 0 to 1. Combining coverage data from scaffolds assembled from different samples prior to normalization made it possible to generate a single ESOM map for binning genomes assembled independently from each sample. ESOMs were trained for 10 epochs using the Somoclu algorithm (35) with the option to initialize the codebook using principal-component analysis (PCA). Genomes were binned by manually selecting data points on the ESOM map using Databionics ESOM Tools (36). Binning was aided by coloring scaffold fragments on the map based on BLAST (37) hits to the genomes assembled in the prior study.

As part of the NIH2 and NIH3 studies, scaffolds were binned based on their GC content, DNA sequence coverage, and taxonomic affiliation using ggKbase tools (ggkbase.berkeley.edu). Genome bins from all three data sets were classified based on the consensus of taxonomic assignments for predicted protein sequences. Genome completeness and contamination were estimated for all genomes using CheckM with the taxonomy_wf option (38). Genomes with extra single-copy genes, but with ≤175 fragments/Mbp (normalized for contamination) that were estimated to be ≥75% complete, were manually curated based on scaffold GC content and coverage.

### Clustering genomes into subspecies groups.

Genomes were clustered into subspecies groups based on sharing ≥98% average nucleotide identity (ANI), as estimated by MASH (39). Representative genomes were selected for each cluster as the largest genome with the highest expected completeness and smallest amount of contamination. Genomes were classified based on the lowest possible consensus of taxonomic assignments for predicted protein sequences.

Taxonomic assignments for representative genomes were checked manually based on hits to ribosomal protein S3 or on visual inspection of protein taxonomic assignments. In order to identify cases in which the same bacterial strain was present in multiple samples, subspecies groups were further analyzed with the ANIm algorithm (40) implemented in dRep (41).

### Measuring microbial community abundance and replication rates.

In order to achieve accurate abundance and replication rate measurements from read mapping, databases of representative genomes were created for each sample. Each database was constructed in order to include a representative genome from important subspecies groups. Priority was given to high-quality draft genome sequences reconstructed from the same sample. Genomes were classified as high-quality drafts based on the requirements for iRep replication rate analysis (https://github.com/christophertbrown/iRep): ≥75% complete, ≤2.5% contamination, and ≤175 scaffolds per Mbp of sequence (21). Genomes were selected to represent subspecies groups using the following priority scheme: (i) high-quality draft genome assembled from the same sample, (ii) high-quality draft genome from the same infant, (iii) high-quality draft genome representative of subspecies group from any infant (if group had ≥5 representatives), and (iv) best genome from infant (if a genome was available). iRep was conducted using reads that mapped to genome sequences with ≤1 mismatch per read sequence. In cases where iRep values were ≥3, coverage plots were inspected and values were removed if there was evidence of strain variation.

We considered a bacterial subspecies to be present in a sample if ≥97% of the genome was covered by an average of ≥2 reads. Abundance and iRep measurements were compared across samples by linking sample-specific representative genomes to subspecies groups. Relative abundance measurements for each subspecies group were calculated by converting DNA sequencing coverage values to a percentage. UniFrac (42) analysis was conducted based on rarefied abundance data, and a tree was constructed based on pairwise genome ANI values measured using MASH (-ms 5000000).

### Metaproteomics analysis.

Metaproteomics sequencing was conducted on 0.3 g of stool as previously described (18). Each sample was suspended in 10 ml cold phosphate-buffered saline. Samples were filtered through a 20-µm-pore-size filter to enrich for microbial cells and proteins. Microbial cells were collected by centrifugation, boiled in 4% sodium dodecyl sulfate for 5 min, and sonicated to lyse cells. The resulting protein extract was precipitated with 20% trichloroacetic acid at −80°C overnight. The protein pellet was washed with ice-cold acetone, solubilized in 8 M urea, and reduced with 5 mM dithiothreitol, and cysteines were blocked with 20 mM iodoacetamide. Then, sequencing-grade trypsin was used to digest the proteins into peptides. Proteolyzed peptides were then salted and acidified by adjusting the sample to 200 mM NaCl–0.1% formic acid followed by filtration through a 10-kDa-cutoff spin column filter to collect tryptic peptides.

Peptides were quantified by bicinchoninic acid (BCA) assay, and 50 µg of peptides of each sample was analyzed via the use of a two-dimensional nanospray liquid chromatography-tandem mass spectrometry (LC-MS/MS) system and an LTQ-Orbitrap Elite mass spectrometer (Thermo Scientific). Each peptide mixture was loaded onto a biphasic back column containing both strong-cation exchange and reverse-phase resins (C_18_). As previously described, loaded peptides were separated and analyzed using an 11-salt-pulse MudPIT protocol over a 22-h period (43). Mass spectra were acquired in data-dependent mode with following parameters: full scans were acquired at 30-K resolution (1 microscan) in the Orbitrap, followed by collision-induced dissociation (CID) fragmentation of the 20 most abundant ions (1 microscan). Charge state screening and monoisotopic precursor selection were enabled. Unassigned charges and charges with a state of +1 were rejected. Dynamic exclusion was enabled with a mass exclusion width of 10 ppm and exclusion duration of 30 s. Two technical replicates were conducted for each sample.

Protein databases were generated for each infant from protein sequences predicted from assembled metagenomes. The database also included human protein sequences (NCBI Refseq_2011), common contaminants, and reverse protein sequences, which were used to control the false-discovery rate (FDR). Collected MS/MS spectra were matched to peptides using MyriMatch v2.1 (44), filtered, and assembled into proteins using IDPicker v3.0 (45). All searches included the following peptide modifications: a static cysteine modification (+57.02 Da), an N-terminal dynamic carbamylation modification (+43.00 Da), and a dynamic oxidation modification (+15.99). A maximum 2% peptide spectrum match level FDR and a minimum of two distinct peptides per protein were applied to achieve confident peptide identifications (FDR of <1%). To alleviate the ambiguity associated with shared peptides, proteins were clustered into protein groups by 100% identity for microbial proteins and 90% amino acid sequence identity for human proteins using USEARCH (26). Spectral counts were balanced between shared proteins, and proteins were considered to be present if ≥2 unique peptides were identified.

### Identification of putative protein families.

Putative protein families were identified in order to track the presence and abundance of different protein types across samples. ORFs were preclustered at 95% identity using USEARCH (-cluster_smallmem -target_cov 0.50 -query_cov 0.95 -id 0.95) first, and then all-versus-all protein searches were conducted (–ublast -evalue 10e-10 -strand both). Protein families were delineated from within the all-versus-all network graph using the MCL clustering algorithm (-I 2 -te 10) (46). The most common annotation observed across all protein sequences in the group was selected as the annotation for the putative protein family. Proteins were also grouped based on sharing 97% amino acid identity using USEARCH (-cluster_smallmem -target_cov 0.50 -query_cov 0.95 -id 0.97).

### Tracking human and bacterial protein abundances.

Human and bacterial protein abundances were normalized using the weighted trimmed mean method from EdgeR (47). Species-specific proteomic profiles were normalized as the percentage of total balanced spectral counts.

### Sample clustering and statistical analyses.

Sample clustering was conducted based on microbial community abundance and iRep profiles and on bacterial protein family abundance profiles. In each case, the number of clusters was determined using the gap statistic (48), and then samples were grouped into the appropriate number of clusters using hierarchical clustering (average linkage method). Microbial community data were clustered based on weighted UniFrac distances, and protein data were clustered using Bray-Curtis distance. EdgeR was used to calculate statistically significant differences between conditions using *quasi*-likelihood linear modeling (glmQLFTest).
