# Supplementary figures and images for: Hospitalized Premature Infants Are Colonized by Related Bacterial Strains with Distinct Proteomic Profiles
Source: mBio. 2018 Apr 10;9(2):e00441-18. doi: 10.1128/mBio.00441-18 (PMC5893878; doi:10.1128/mBio.00441-18)

# Supplemental Figure 1

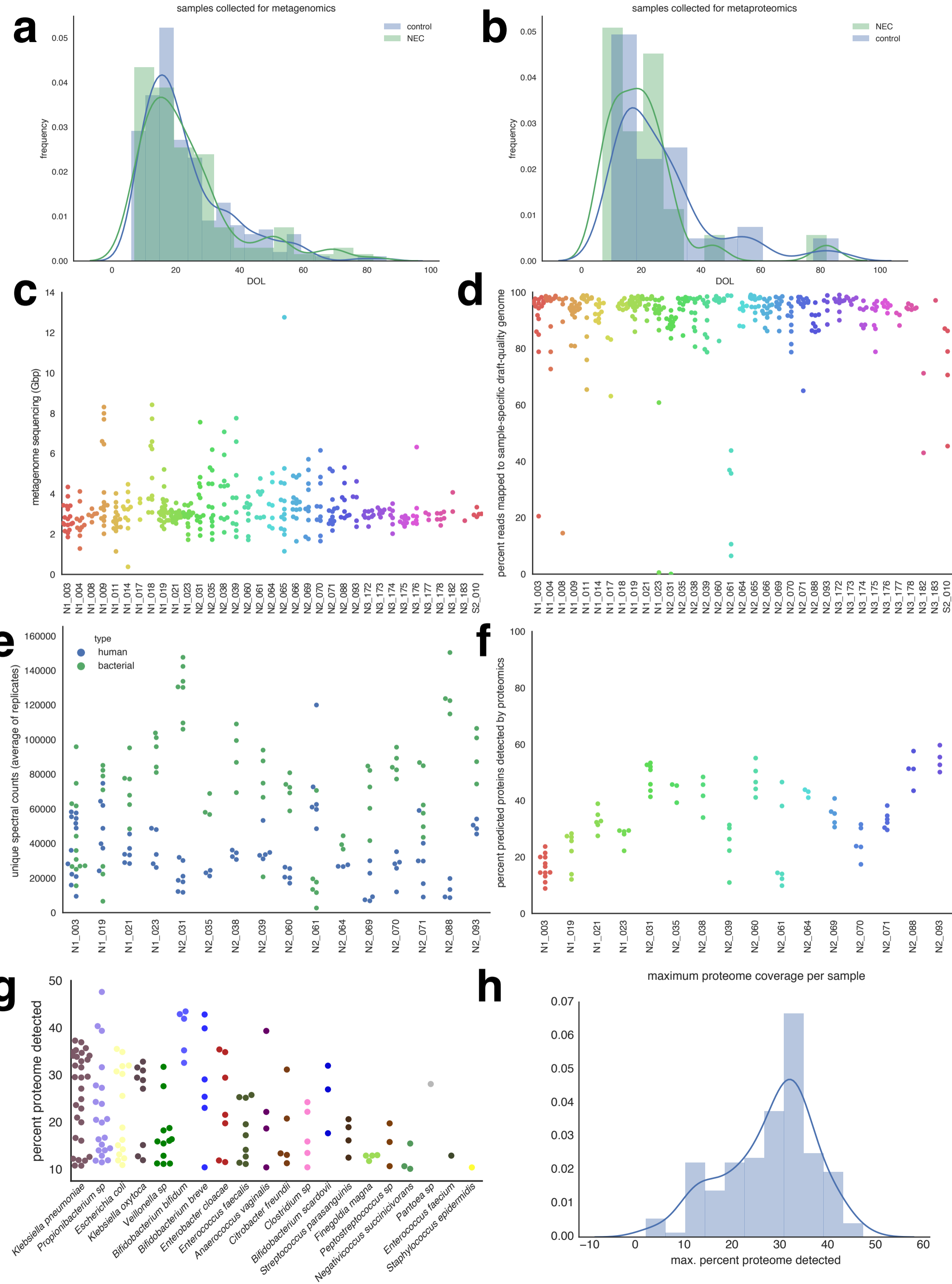

Supplement: FIG S1 [file mbo002183830sf1.pdf]

# Supplemental Figure 2

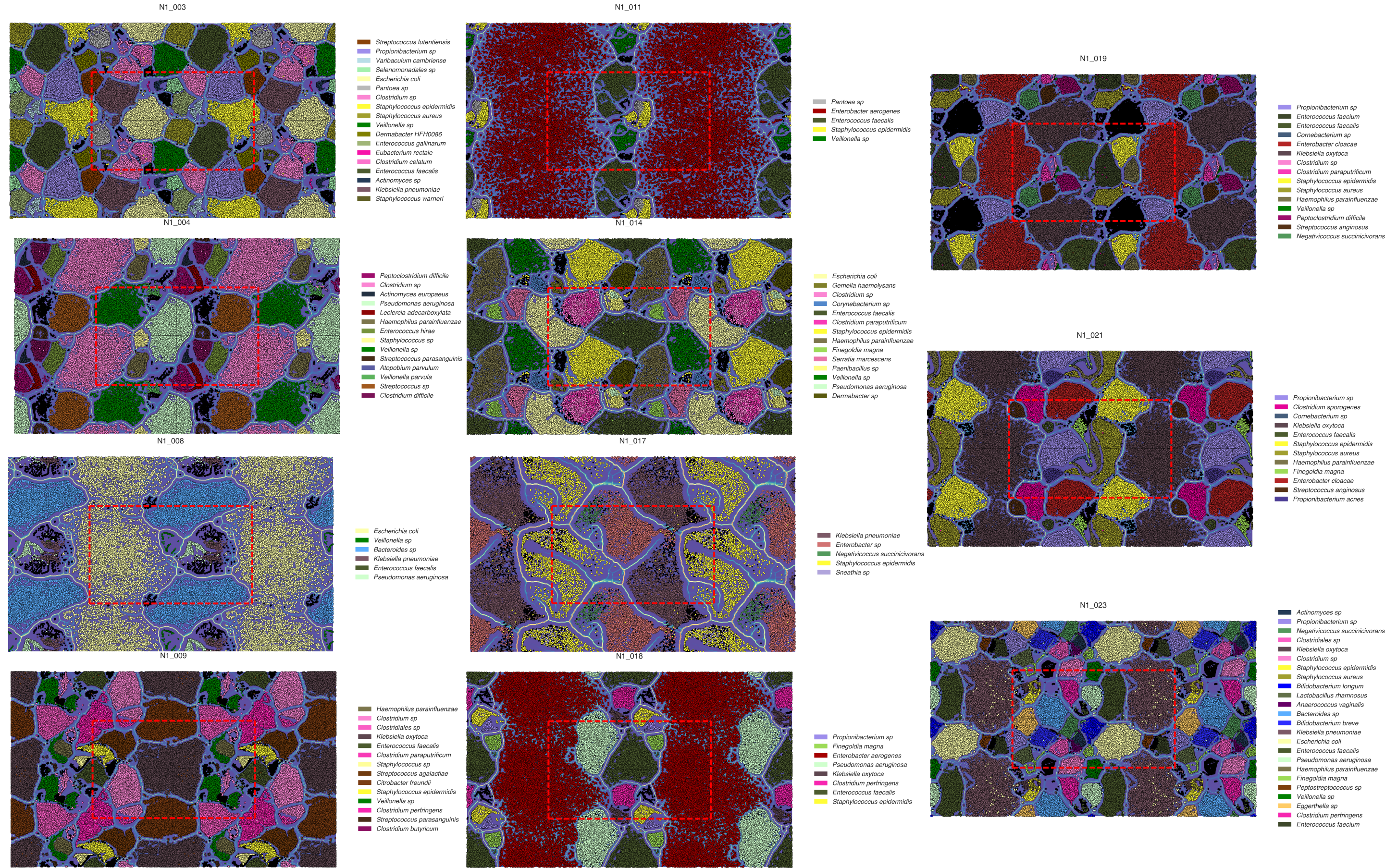

Supplement: FIG S2 [file mbo002183830sf2.pdf]

# Supplemental Figure 3

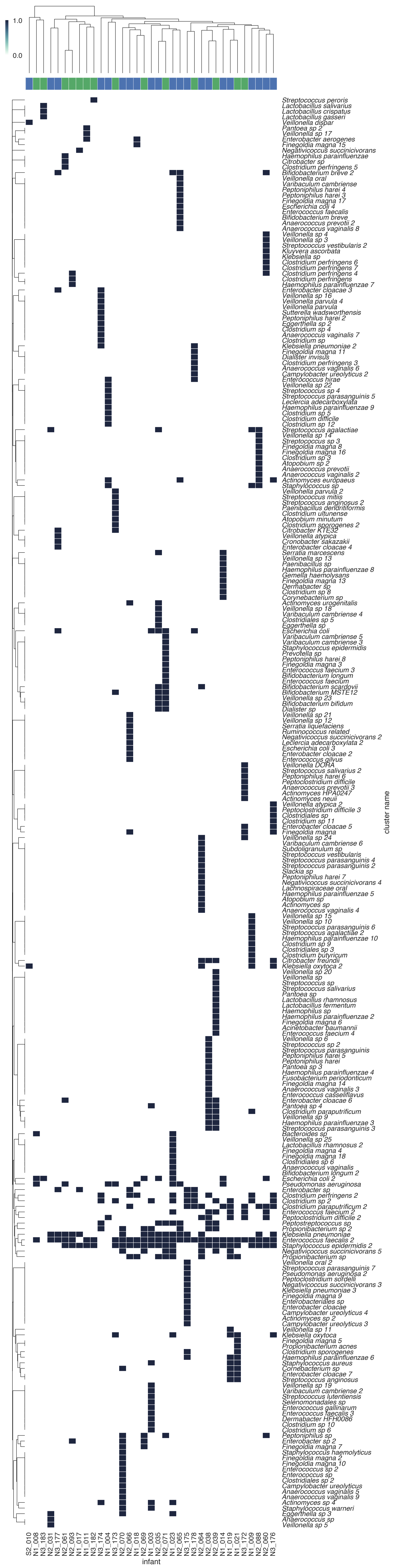

Supplement: FIG S3 [file mbo002183830sf3.pdf]

# Supplemental Figure 4

a

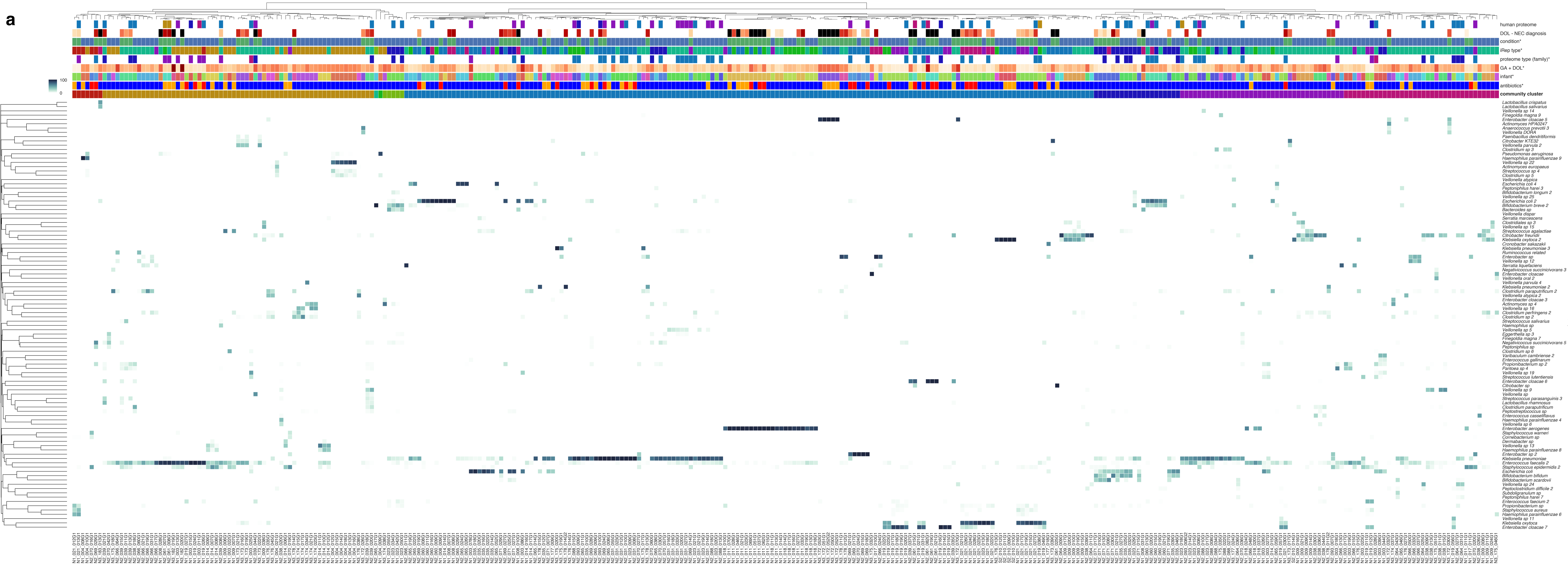

b

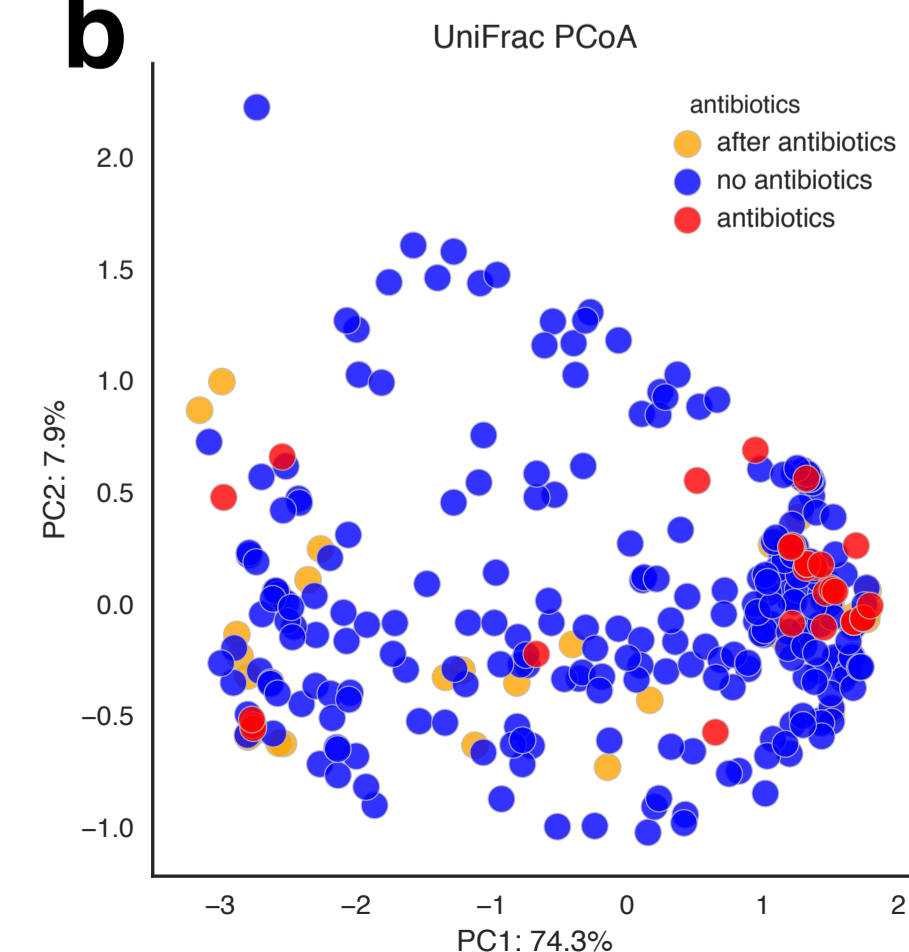

c

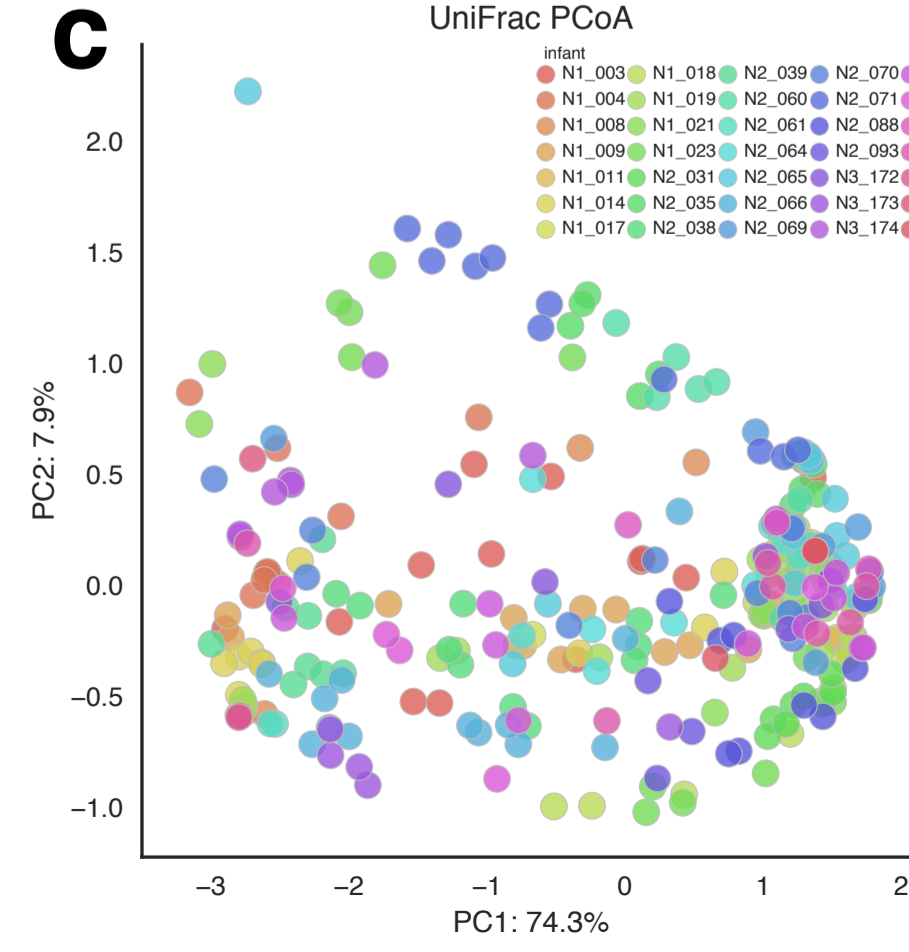

d

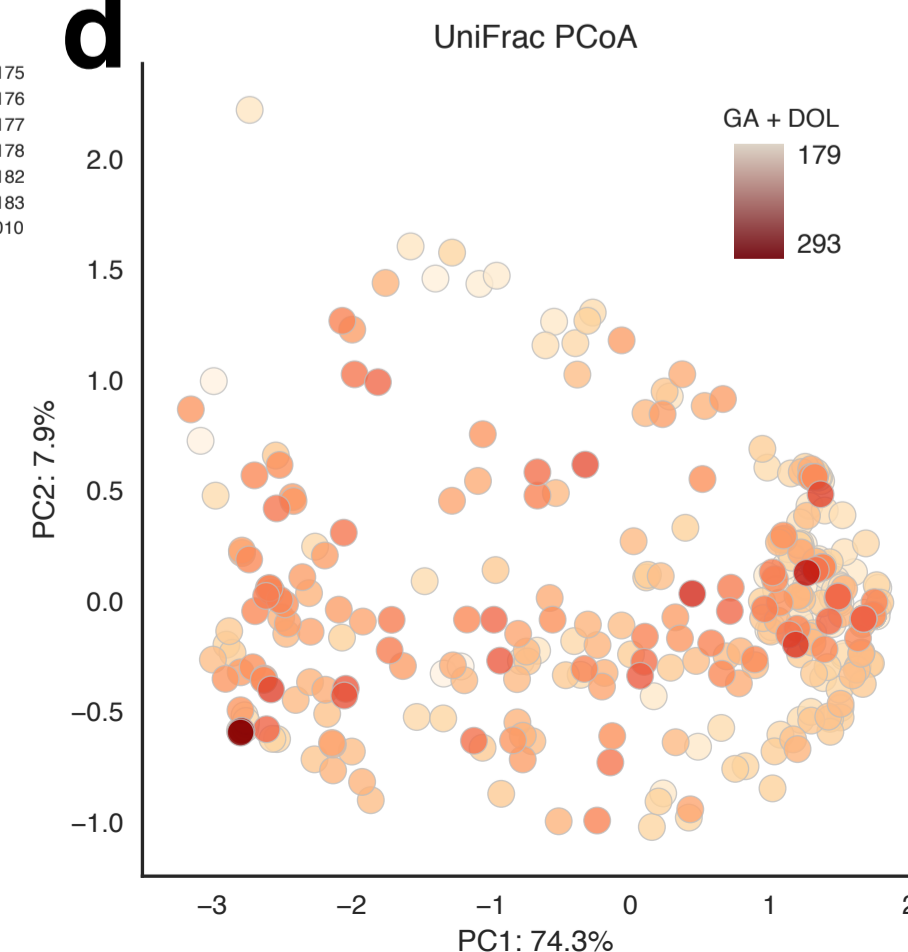

e

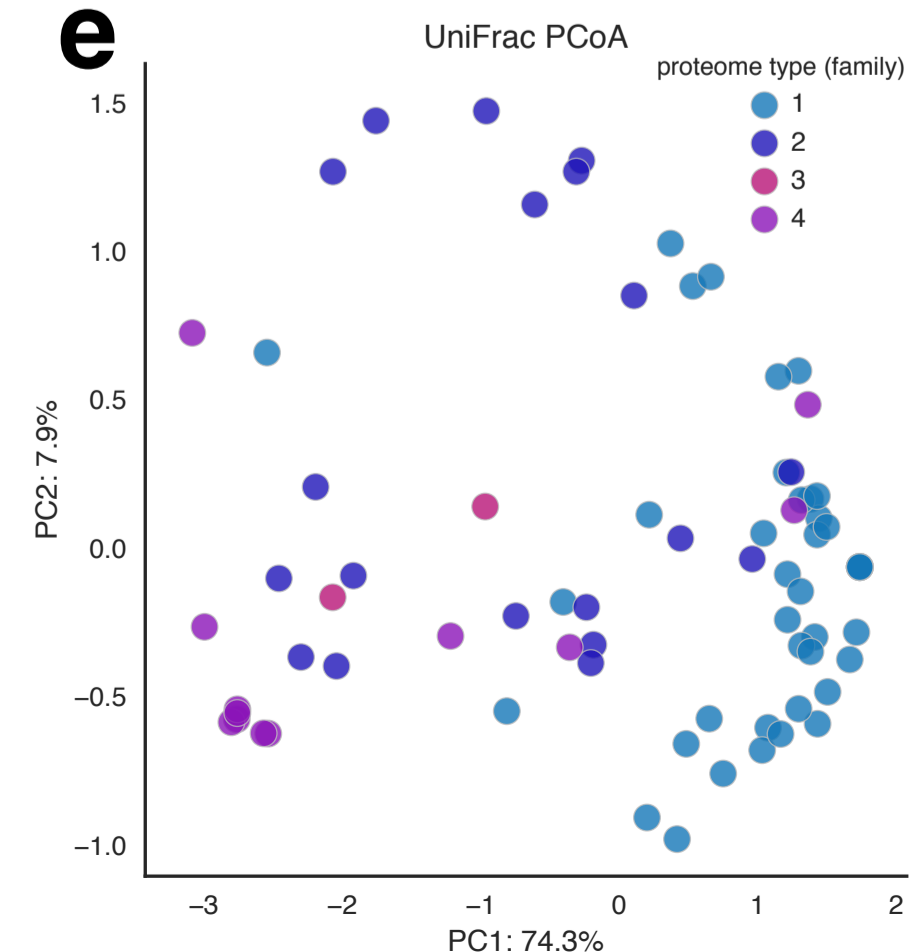

f

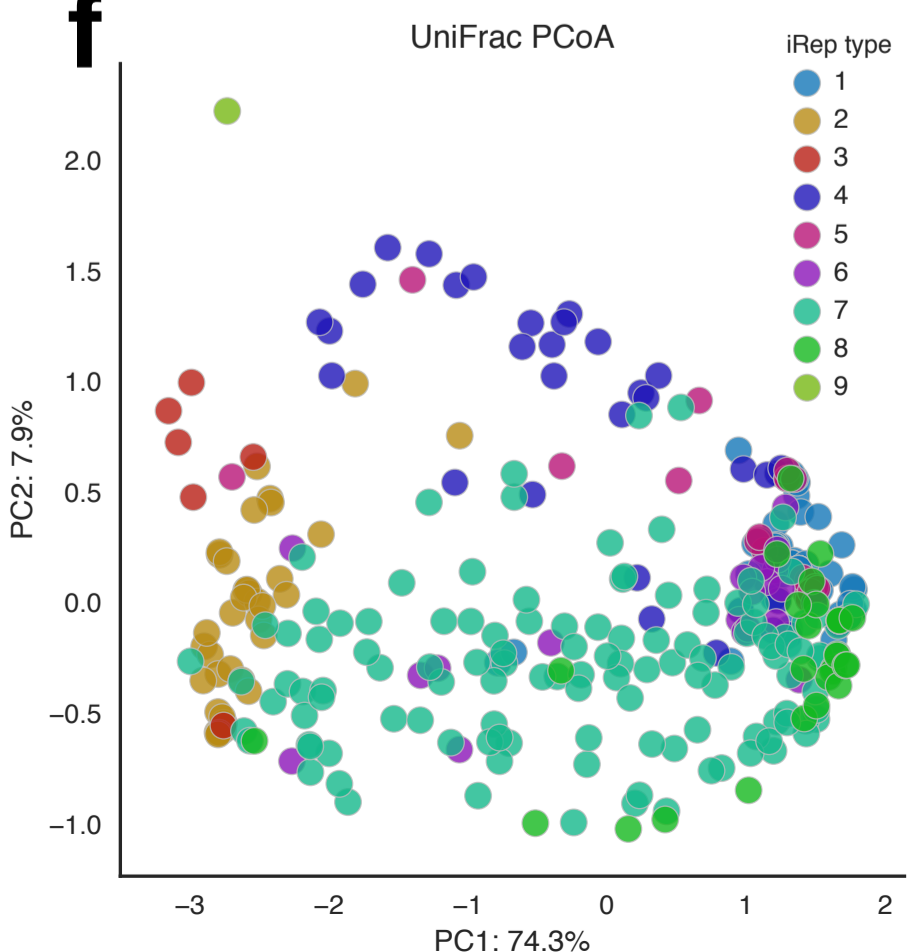

g

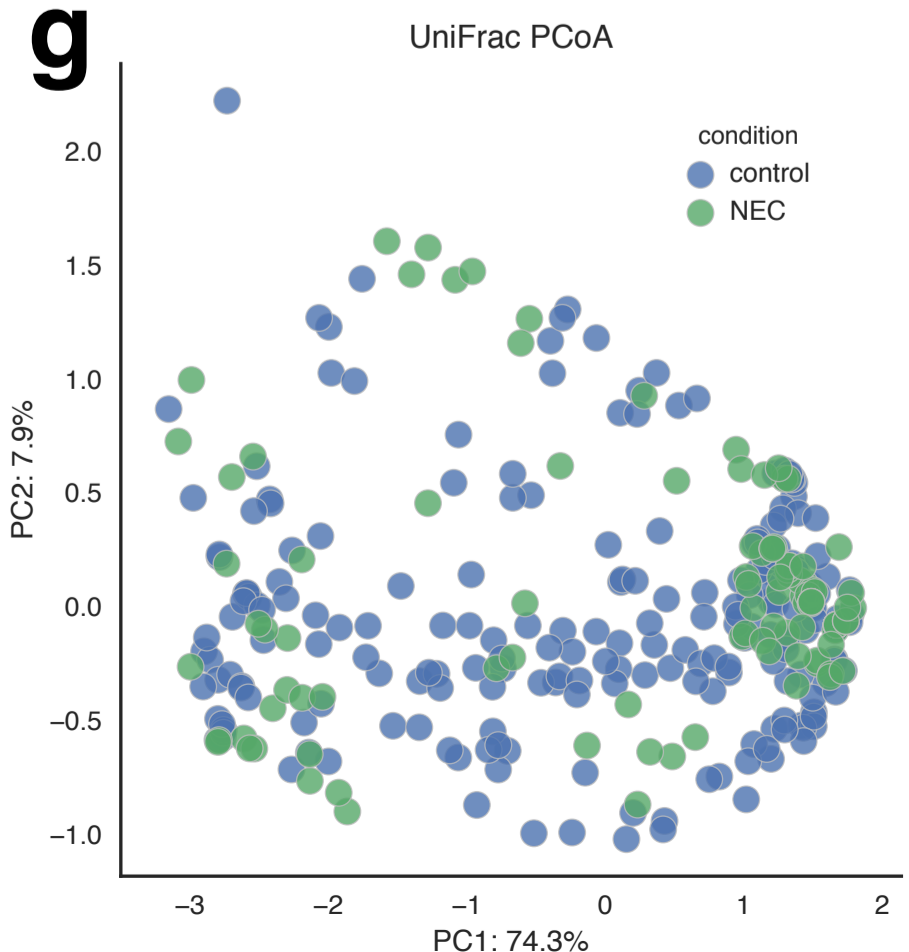

h

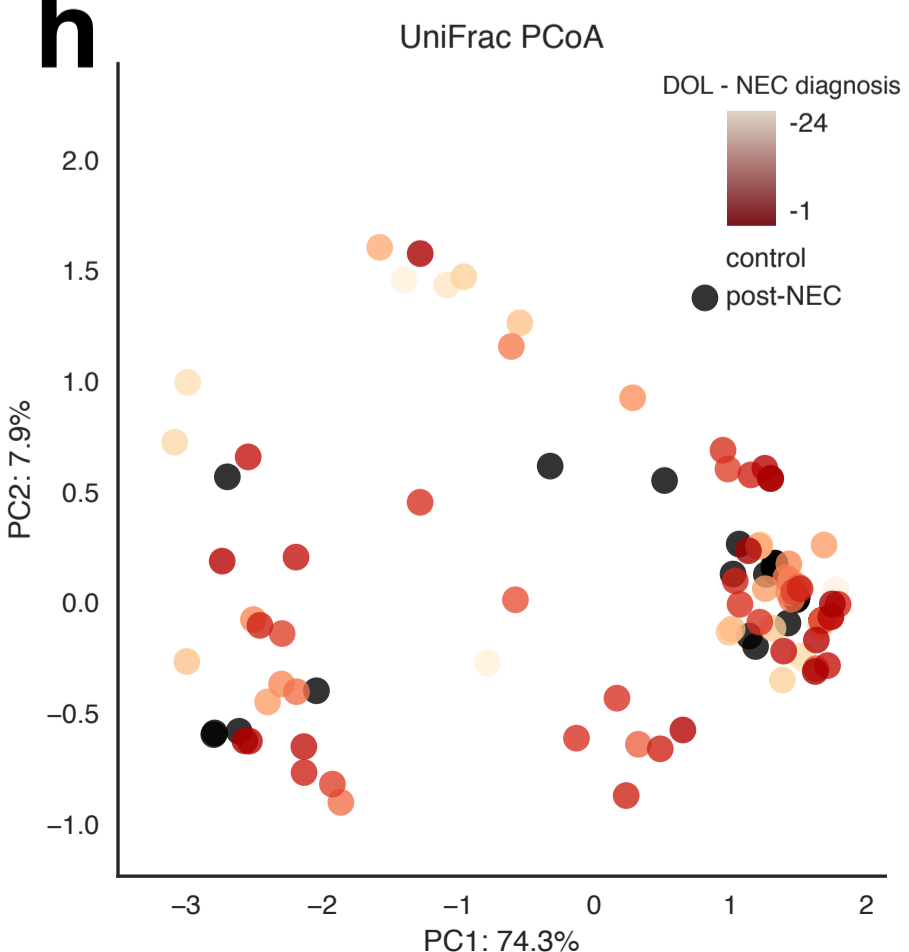

i

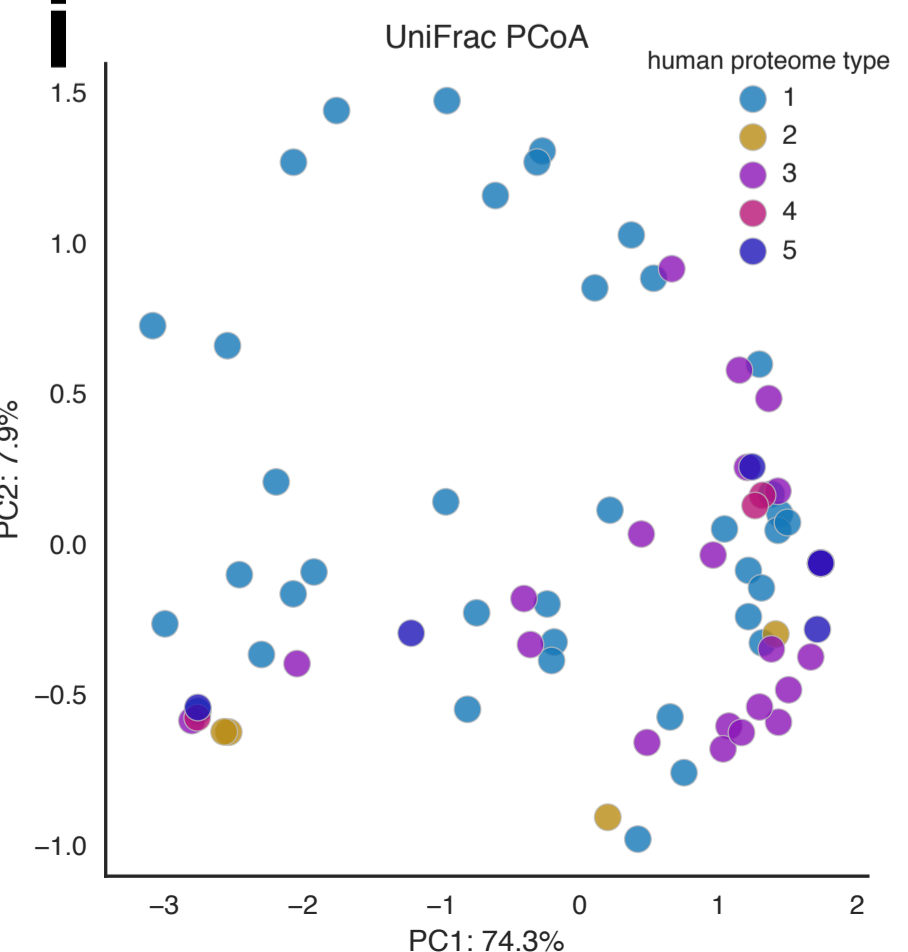

Supplement: FIG S4 [file mbo002183830sf4.pdf]

## Supplemental Figure 5

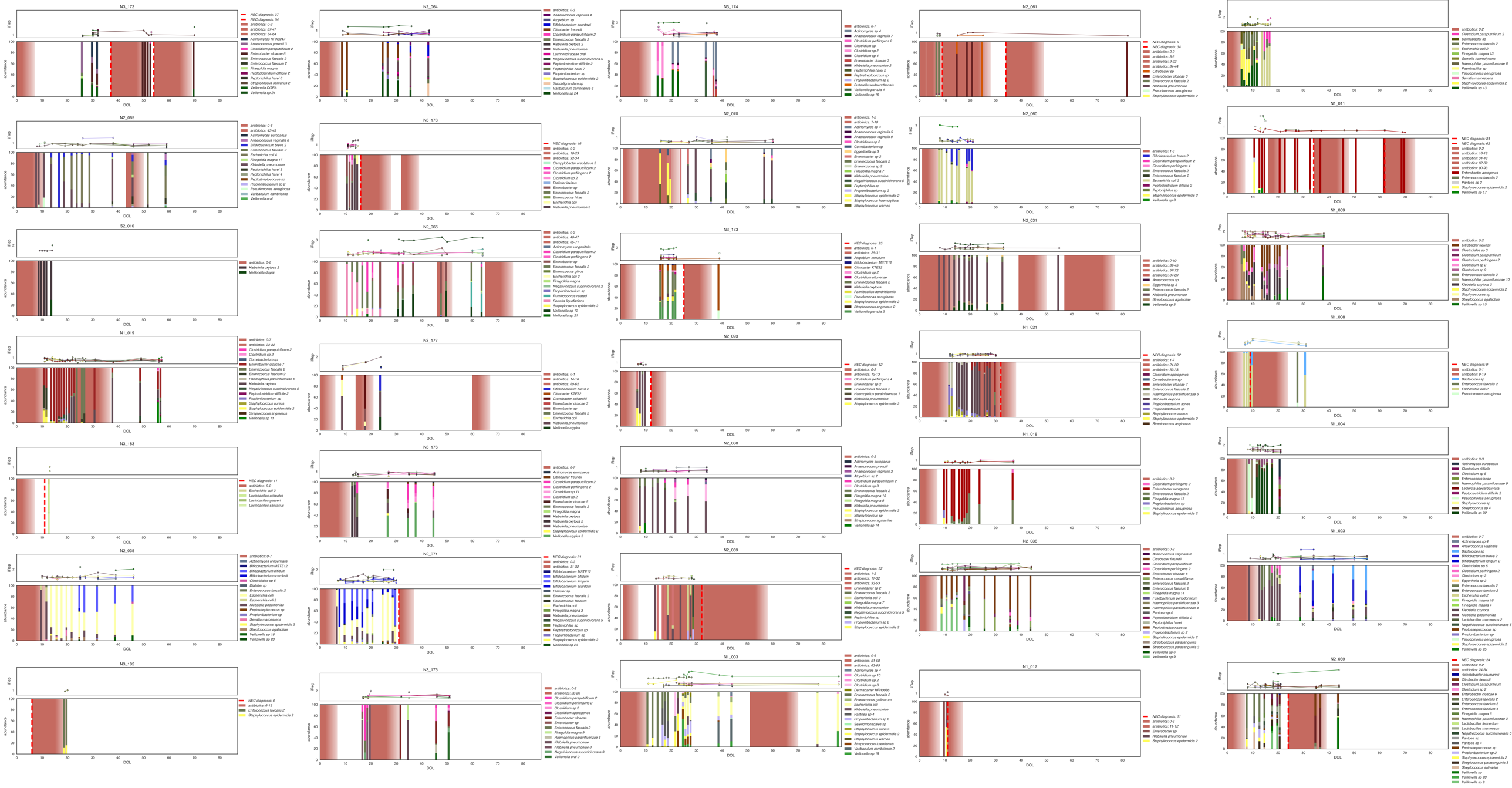

Supplement: FIG S5 [file mbo002183830sf5.pdf]

# Supplemental Figure 6

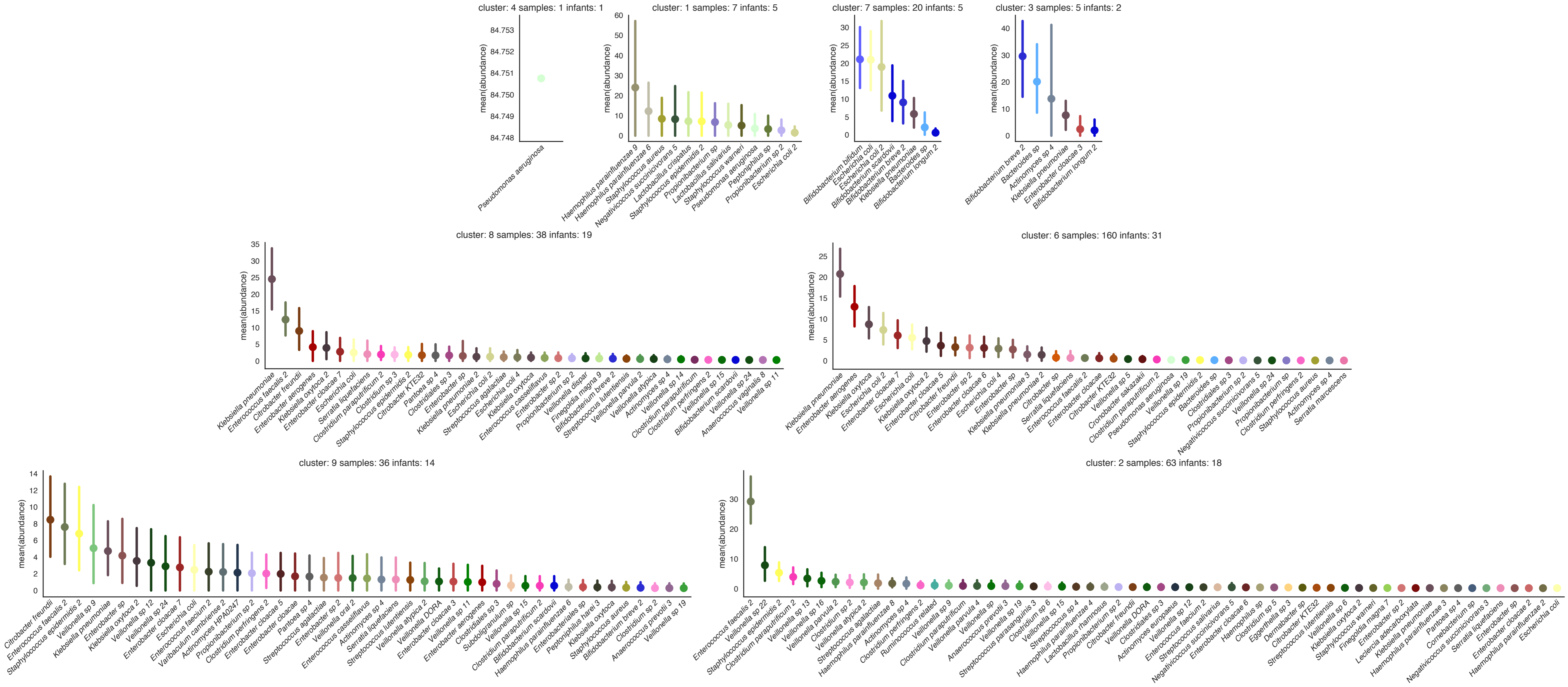

Supplement: FIG S6 [file mbo002183830sf6.pdf]

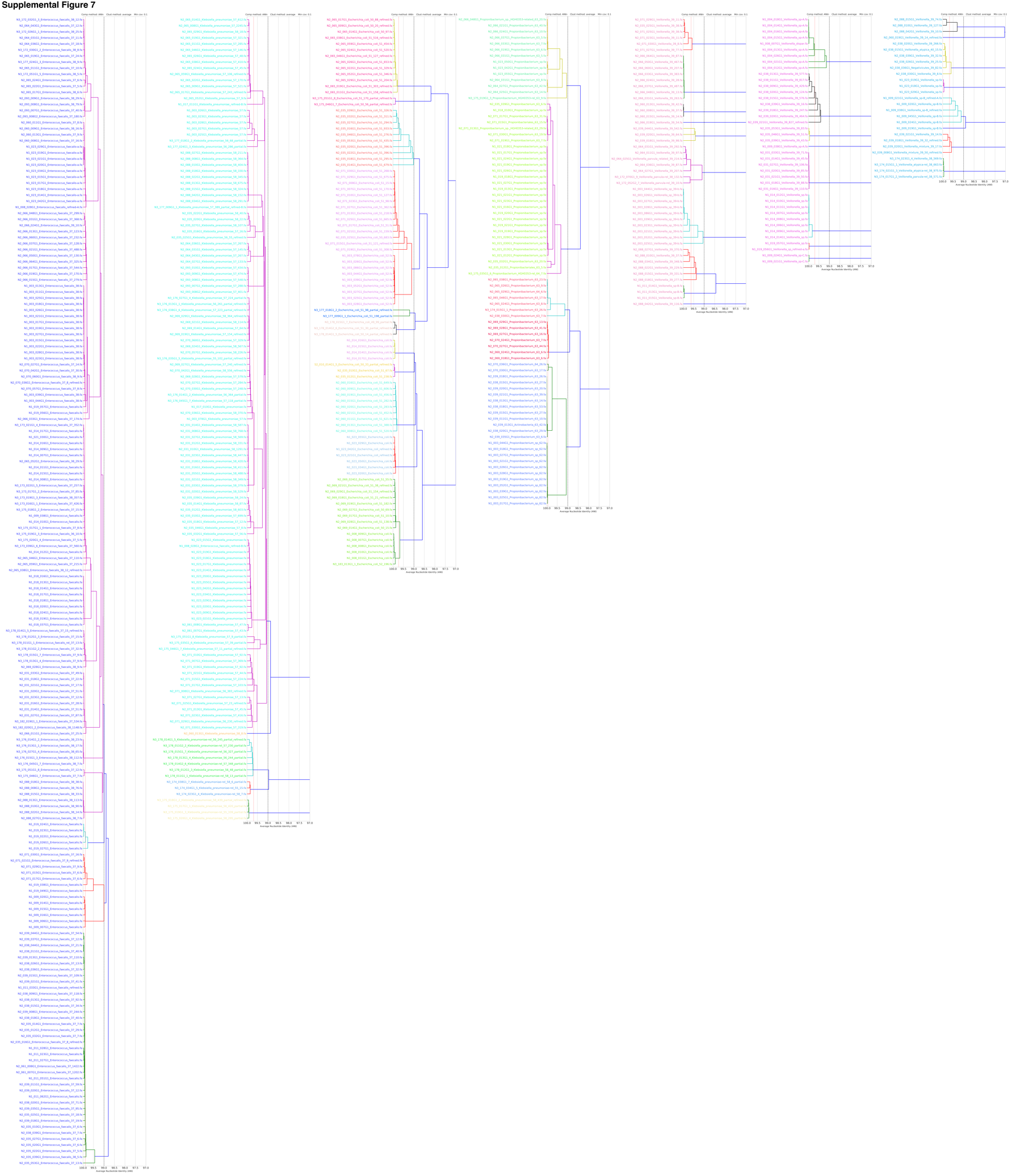

Supplement: FIG S7 [file mbo002183830sf7.pdf]

# Supplemental Figure 8

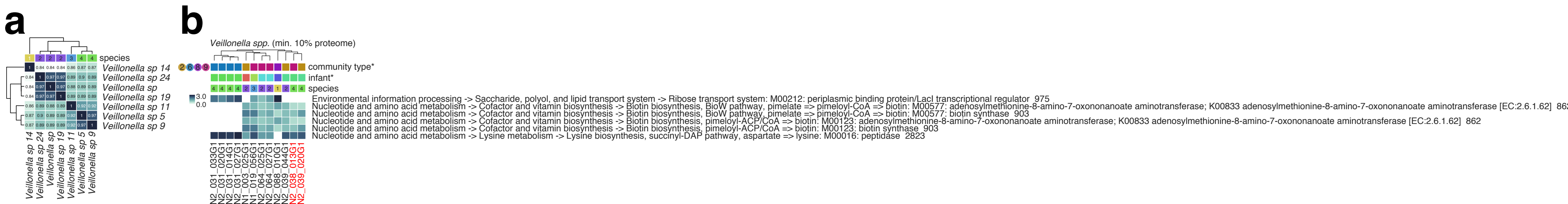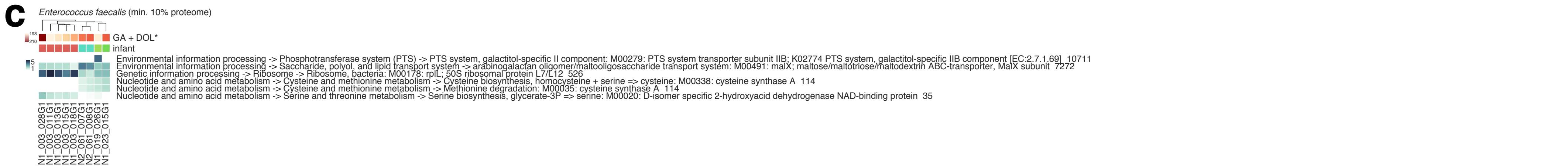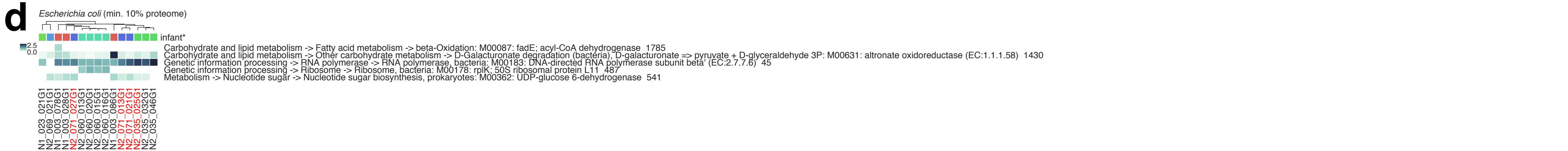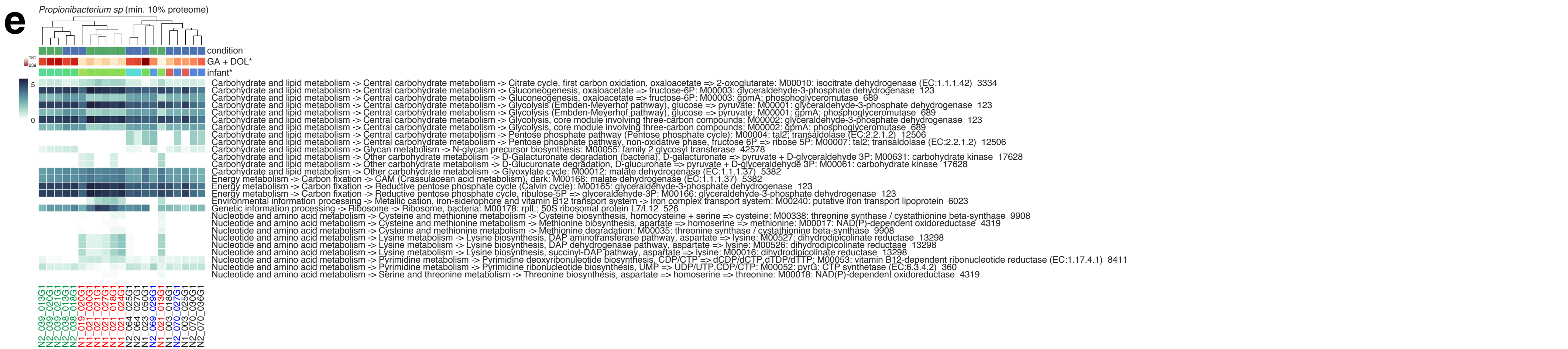

Supplement: FIG S8 [file mbo002183830sf8.pdf]

# Supplemental Figure 9

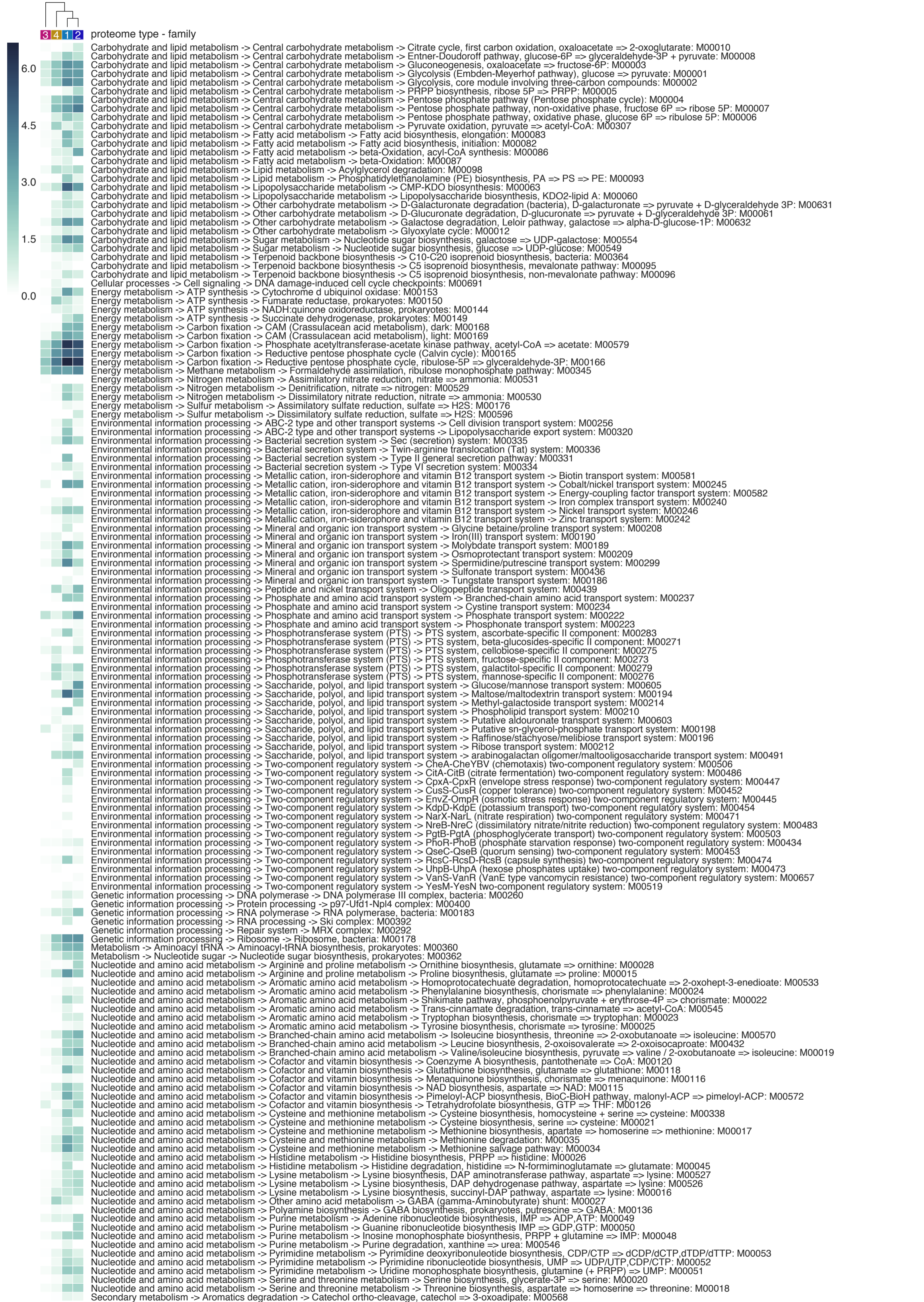

Supplement: FIG S9 [file mbo002183830sf9.pdf]
